# A mouse model for distal renal tubular acidosis reveals a previously unrecognized role of the V-ATPase a4 subunit in the proximal tubule

**DOI:** 10.1002/emmm.201201527

**Published:** 2012-08-30

**Authors:** J Christopher Hennings, Nicolas Picard, Antje K Huebner, Tobias Stauber, Hannes Maier, Dennis Brown, Thomas J Jentsch, Rosa Vargas-Poussou, Dominique Eladari, Christian A Hübner

**Affiliations:** 1 Institut für Humangenetik, Universitätsklinikum JenaJena, Germany; 2 Faculté de Médecine, Université Paris-DescartesSorbonne Paris-Cité, Paris, France; 3 INSERM UMRS 872, Centre de Recherche des CordeliersParis, France; 4 Leibniz-Institut für Molekulare Pharmakologie (FMP) and Max-Delbrück Centrum für Molekulare Medizin (MDC)Berlin, Germany; 5 Institut für Audioneurotechnologie und Abteilung für Experimentelle Ohrenheilkunde der Klinik für Hals-Nasen-Ohrenheilkunde, Medizinische Hochschule HannoverHannover, Germany; 6 Program in Membrane Biology, Division of Nephrology, Center for Systems Biology, Massachusetts General Hospital, Harvard Medical School, Simches Research CenterBoston, USA; 7 Hopital Européen Georges Pompidou, Service de Génétique, Assistance Publique-Hopitaux de ParisParis, France; 8 Département de Physiologie, Hopital Européen Georges Pompidou, Assistance Publique-Hopitaux de ParisParis, France

**Keywords:** deafness, distal renal tubular acidosis, H^+^-ATPase, kidney, proteinuria

## Abstract

The V-ATPase is a multisubunit complex that transports protons across membranes. Mutations of its B1 or a4 subunit are associated with distal renal tubular acidosis and deafness. In the kidney, the a4 subunit is expressed in intercalated cells of the distal nephron, where the V-ATPase controls acid/base secretion, and in proximal tubule cells, where its role is less clear. Here, we report that a4 KO mice suffer not only from severe acidosis but also from proximal tubule dysfunction with defective endocytic trafficking, proteinuria, phosphaturia and accumulation of lysosomal material and we provide evidence that these findings may be also relevant in patients. In the inner ear, the a4 subunit co-localized with pendrin at the apical side of epithelial cells lining the endolymphatic sac. As a4 KO mice were profoundly deaf and displayed enlarged endolymphatic fluid compartments mirroring the alterations in pendrin KO mice, we propose that pendrin and the proton pump co-operate in endolymph homeostasis. Thus, our mouse model gives new insights into the divergent functions of the V-ATPase and the pathophysiology of a4-related symptoms.

## INTRODUCTION

**Figure 1:**
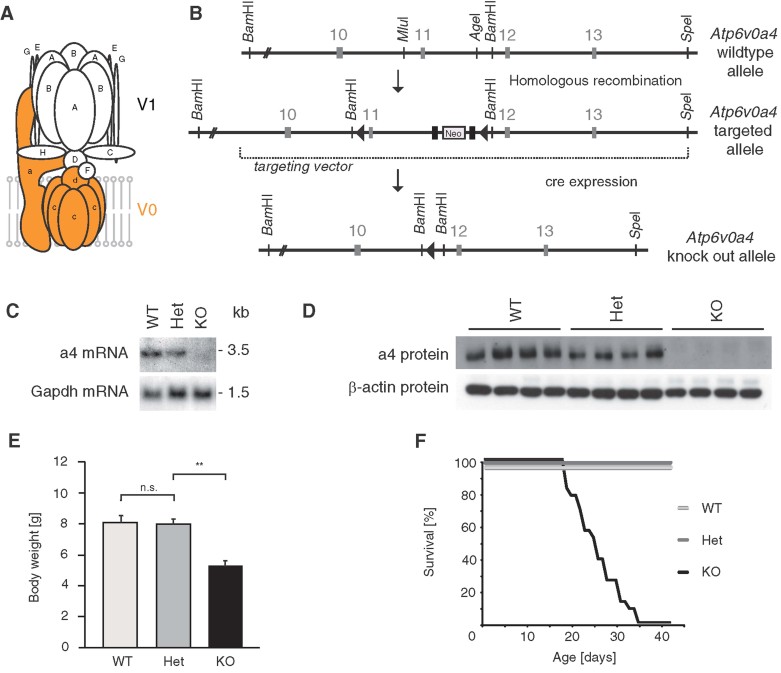
Disruption of the a4 subunit in mice results in early mortality Cartoon of the V-ATPase with its transmembrane (V0) and peripheral (V1) sector, each build up by different subunits. The V0 sector including the a subunit is highlighted in orange.Partial genomic structure of the *Atp6v0a4* gene (top) and the targeted a4 locus. The dotted line indicates the genomic sequence included in the targeting construct. A neomycin selection cassette flanked by frt sites (black boxes) and single loxP site was inserted into intron 11. A second loxP site and a *Bam*HI site were introduced into intron 10. Correctly targeted ES cell clones were used for the generation of chimeric mice (below). a4 KO mice were generated by breeding chimeric mice to a cre-deleter mouse strain (bottom).a4 transcript abundance in kidneys of *Atp6v0a4*^+/+^ (WT), *Atp6v0a4*^+/−^ (Het) and *Atp6v0a4*^−/−^ (KO) mice as revealed by Northern blot. Gapdh mRNA served as a loading control.Detection of the a4 subunit by Western blot analysis of kidney protein lysates of four individual mice per genotype; β-actin served as a loading control.Significant reduction of body weight in *Atp6v0a4*^−/−^ mice at 3 weeks of age (**p < 0.01).Kaplan–Meier plot during early postnatal life. *Atp6v0a4*^+/+^ (*n* = 25) and *Atp6v0a4*^+/−^ (*n* = 40) are viable. *Atp6v0a4*^−/−^ mice (*n* = 23) have an increased mortality beginning at 19 days of age.

The V-ATPase is a multisubunit enzyme that uses the energy derived from the hydrolysis of cytosolic ATP to translocate protons across biological membranes. It consists of a catalytic V1 domain for ATP hydrolysis and a transmembrane V0 domain that mediates proton translocation (Fig 1A; Forgac, 2007; Marshansky & Futai, 2008; Wagner et al, 2004). V1 and V0 can exist separately, but must combine in order to pump protons. The cytosolic V1 domain consists of the subunits A–H (denoted in capital letters), and the membrane-bound V0 domain of subunits a, d and c with several copies of the c subunit. For some subunits, there are various isoforms and the subunit composition can differ considerably between various sites of expression. Even in any single cell type, different isoforms of a particular subunit can be present in various subcellular structures (Marshansky & Futai, 2008).

In most cell types, V-ATPase expression is restricted to intracellular membranes where it mediates acidification of the lumen of intracellular organelles including endosomal and lysosomal compartments. However, in some highly specialized cells such as osteoclasts (Lee et al, 1996), epididymal clear cells (Shum et al, 2009) or renal intercalated cells (ICs; Wagner et al, 2004) the V-ATPase is also expressed at the plasma membrane. In these cells, the V-ATPase mediates acidification of the extracellular space, a process critical for bone resorption, male fertility and renal acid secretion.

A defect of distal acid secretion is the leading symptom in autosomal recessive distal renal tubular acidosis (dRTA) and can be caused by mutations in the genes encoding either the ATP6V0B1 (B1) or the ATP6V0A4 (a4) subunit of the V-ATPase (Karet et al, 1999; Smith et al, 2000). Whereas in mammals two isoforms exist for the B subunit, which is part of the V1 domain, four different isoforms are known for the a subunit, which is an integral membrane protein of the V0 domain (Forgac, 2007). Some patients carrying mutations in either the *ATP6V0A4* or the *ATP6V1B1* gene also suffer from hearing impairment of variable degree (Stover et al, 2002; Vargas-Poussou et al, 2006).

Both subunits are heavily expressed in ICs (Schulz et al, 2007), which are found in renal connecting tubules as well as collecting ducts and are the main effectors of the fine regulation of renal acid/base homeostasis (Kim et al, 1999b). Whereas type A-ICs secrete acid apically via the V-ATPase and recover bicarbonate basolaterally via the Na^+^-independent anion-exchanger 1 (Ae1, Slc4a1), type B-ICs secrete bicarbonate apically via the Na^+^-independent anion-exchanger pendrin (Pds, Slc26a4) and express the V-ATPase at their basolateral side (Alper et al, 1989; Brown et al, 1988; Royaux et al, 2001).

In contrast to the B1 subunit, the a4 subunit is also expressed in proximal tubule cells, where it has been localized both to the apical plasma membrane and to intracellular vesicles (Hurtado-Lorenzo et al, 2006; Stehberger et al, 2003). The proximal tubule is equipped with a highly specialized machinery for the absorption of many different substrates from the primary filtrate and thus critically contributes to the homeostasis of the milieu interne. Filtered proteins are recovered in the proximal tubule by endocytosis and are subsequently degraded within lysosomes, a process that has been shown to depend on the V-ATPase (Marshansky et al, 2002; Marshansky & Futai, 2008). Defective endosomal acidification has been suggested to underlie the proteinuria and hypercalcinuria of Dent's disease (Günther et al, 2003; Piwon et al, 2000), although recent data also implicate changes in endosomal Cl^−^ concentration (Novarino et al, 2010).

Whereas a knockout mouse model for the B1 subunit did not display overt metabolic acidosis or hearing loss (Dou et al, 2003; Finberg et al, 2005), we show here that the disruption of the a4 subunit causes a fatal phenotype with severe dRTA and deafness. We further show that dRTA patients carrying mutations in the a4 subunit were also more severely affected than dRTA patients related to the B1 subunit. The disruption of the a4 subunit also perturbed proximal tubule function resulting in phosphaturia, proteinuria and the accumulation of lysosomal material in proximal tubule cells. As we provide evidence that these observations may contribute to the complex renal phenotype of at least some patients with mutations in the a4 subunit, our findings may require a revision of the current dogma that kidney disease in dRTA arises only from defects in the distal tubule.

## RESULTS

### Disruption of Atp6v0a4 in mice results in a severe phenotype with early mortality

To disrupt the *Atp6v0a4* gene in mice, we deleted a fragment including exon 11 by loxP/Cre-mediated recombination (Fig 1B). At birth, heterozygous (*Atp6v0a4*^+/−^, Het) and homozygous (*Atp6v0a4*^−/−^, KO) knockout mice appeared indistinguishable from their wildtype (*Atp6v0a4*^+/+^, WT) littermates and were born from heterozygous matings at Mendelian ratio. Northern blot analysis of *Atp6v0a4* transcript abundance of total RNA isolated from P21 kidneys from *Atp6v0a4*^−/−^ mice suggested that the variant transcript was subjected to nonsense-mediated RNA decay (Fig 1C). The absence of the a4 protein from *Atp6v0a4*^−/−^ kidney lysates was confirmed by Western blot analysis (Fig 1D). Homozygous knockout mice failed to thrive resulting in a lower body weight (5.44 ± 0.26 g, *n* = 5) compared to WT (8.06 ± 0.50 g, *n* = 5; *p* < 0.01) and heterozygous littermates (7.97 ± 0.32 g, *n* = 6; *p* < 0.01; Fig 1E), and died within the first 3–5 weeks of life (Fig 1F). As described earlier, immunostainings for the a4 subunit labelled both proximal tubules as well as cortical and medullary collecting ducts (Supporting Information Fig S1A–C). At the light microscopy level, no gross alterations of the kidney of *Atp6v0a4*^−/−^ mice were detected (Supporting Information Fig S2A–D).

### Atp6v0a4 deficiency leads to severe dRTA

**Figure 2:**
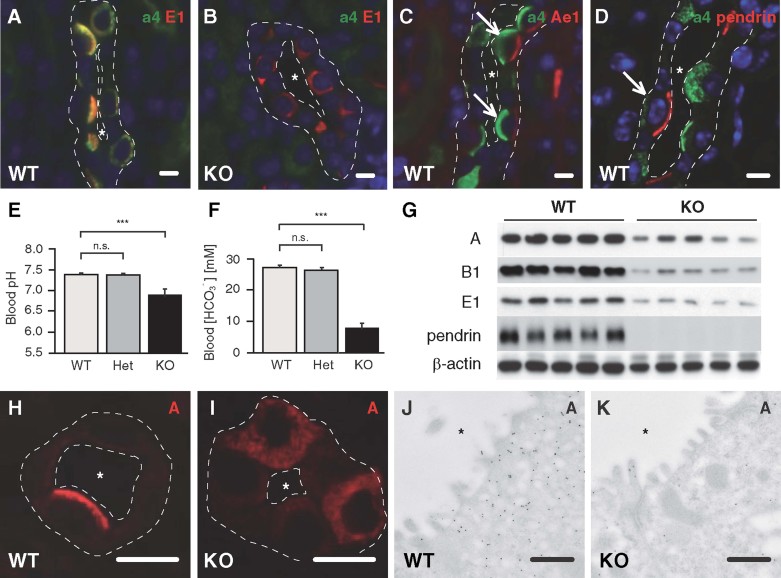
Localization of the a4 subunit in the distal tubule and metabolic acidosis in a4 KO mice For clarity the basolateral and apical borders of the tubular epithelium are indicated by dotted lines and the lumina are marked with an asterisk in immunofluorescence images. **A.** Co-localization of the a4 (green) and E1 subunit of the V-ATPase (red) in ICs from the cortical collecting duct of a WT kidney section. Scale bar: 10 µm.**B.** Loss of targeting of the E1 subunit (red) to the apical cell pole in an a4 KO mouse kidney section. Scale bar: 10 µm.**C,D.** The a4 subunit (green) is expressed in Ae1-positive (red) type A- and pendrin-positive (red) type B-ICs. Scale bars: 10 µm.**E,F.** Severe metabolic acidosis in a4 KO mice as shown by a decrease of the systemic blood pH and [HCO_3_^−^] concentration. For plotted values refer to Table 1. ***p < 0.001.**G.** Western Blot analysis of kidney lysates from five individual mice per genotype shows a decrease of the A, B1, E1 subunit and pendrin expression in a4 KO mice; β-actin served as a loading control.**H,I.** The apical targeting of the A subunit (red) is lost in the a4 KO. Scale bars: 10 µm.**J,K.** Immunogold staining for the A subunit revealed considerably less V-ATPase at the apical plasma membrane and in apically localized intracellular vesicles of ICs of the a4 KO mouse. The asterisk indicates the luminal side. Scale bars: 500 nm.

In the collecting duct, the a4 subunit was expressed in ICs, where it co-localized with the E1 subunit of the V-ATPase (Fig 2A). The lack of labelling in KO kidney sections showed that the a4 antibody was specific (Fig 2B). In acid-secreting type A-ICs that were identified by their basolateral labelling for Ae1, the a4 staining was apical (Fig 2C), whereas it was basolateral in bicarbonate-secreting type B-ICs, which were labelled for pendrin (Fig 2D). In contrast to a previous report (Stehberger et al, 2003), we did not detect any specific labelling of the thick ascending limb or the connecting tubule (Supporting Information Fig S1A–C).

In humans, metabolic acidosis is defined by a blood pH < 7.38 associated with a decrease in plasma [HCO_3_^−^] (Morris & McSherry, 1972). In line with a major defect of renal proton secretion, blood gas analysis in *Atp6v0a4*^−/−^ mice revealed a systemic pH of 6.89 ± 0.20 and a drastic reduction of plasma [HCO_3_^−^], whereas *Atp6v0a4*^+/−^ mice exhibited a normal acid–base status like their WT littermates (Fig 2E–F and Table 1). Under the stimulus of systemic acidosis as observed in a4 KO mice, intact distal acid secretion should lead to maximally decreased urine pH values below pH 5.5. Hence, the urine pH of 6.69 in a4 KO mice suggests a severe distal acidification defect. *Atp6v0a4*^+/+^ and *Atp6v0a4*^+/−^ mice could efficiently secrete acid into the urine in response to metabolic acidification by adding 0.28 M NH_4_Cl to the drinking water (Supporting Information Fig S3A–B), suggesting that the failure of *Atp6v0a4*^−/−^ mice to increase the net acid/ammonium excretion reflected the absolute requirement of the a4 subunit for normal urine acidification but not the functional immaturity of the kidney at that age.

**Table 1:** Blood and urine data from 21-day-old pups

	*Atp6v0a4* genotype		
	
	WT	Het	KO
Blood			
pH	7.39 ± 0.02 **(5)**	7.37 ± 0.02 **(6)**	6.89 ± 0.20 (5)***
pCO_2_ (mmHg)	47 ± 2 **(5)**	49 ± 2 **(6)**	35 ± 5 **(5)**^n.s.^
HCO_3_^−^ (mM)	27.1 ± 0.6 **(5)**	26 ± 0.4 **(6)**	7.6 ± 1.6 (5)***
pO_2_ (mmHg)	42 ± 4 **(5)**	41 ± 3 **(6)**	95 ± 18 (5)*
Na^+^ (mM)	141 ± 1**(5)**	142 ± 1 **(6)**	149 ± 1 (5)**
K^+^ (mM)	7.66 ± 0.82 **(5)**	7.63 ± 0.58 **(6)**	4.38 ± 0.23 (5)**
Cl^−^ (mM)	112 ± 1 **(5)**	113 ± 1 **(6)**	128 ± 3 (5)***
Ca^2+^ (mM)	1.35 ± 0.02 **(5)**	1.36 ± 0.02 **(6)**	1.770 ± 0.04 (5)***
Urine			
pH	7.07 ± 0.24 **(8)**	6.66 ± 0.21 **(13)**	6.69 ± 0.07 (7)*
UNH_4_^+^/UCrea	3.2 ± 1.2 **(7)**	5.4 ± 1.4 **(12)**	7.8 ± 1.7 **(8)**^n.s.^
UNa^+^/UCrea	13 ± 3 **(7)**	9 ± 2 **(12)**	14 ± 6 **(6)**^n.s.^
UK^+^/UCrea	109 ± 27 **(7)**	55 ± 6 **(12)** §	54 ± 10 **(6)**^n.s.^
UP_i_/Ucrea	2.2 ± 0.6 **(10)**	0.7 ± 0.1 **(6)**	13.9 ± 3.7 (8)**

Physiological blood and urine parameters in *Atp6v0a4*^*+/+*^ (WT), *Atp6v0a4*^*+/−*^ (Het) and *Atp6v0a4*^*−/−*^ (KO) littermates on a standard diet.

Values are means ± SE measured on blood or spot urine; values in bold parentheses are the number of mice studied (n.s., not significant; *p < 0.05, **p < 0.01, ***p < 0.001).

We next tested whether an adaptive response to metabolic acidosis of the collecting duct could be observed in the absence of the a4 subunit in order to increase proton secretion. Normally the V-ATPase is translocated from the cytoplasm to the apical plasma membrane of type A-ICs upon acidosis, whereas it is translocated from the basolateral plasma membrane to the cytoplasm in type B-ICs (Schwartz et al, 1985). In *Atp6v0a4*^−/−^ mice, however, the immunostaining on tissue sections for the E1 (Fig 2B and Supporting Information Fig S4D) and the A subunit (Fig 2I and Supporting Information Fig S4B) was diffusely cytoplasmic, in striking contrast to the staining of the apical cell pole observed in *Atp6v0a4*^+/+^ mice (Fig 2A and H and Supporting Information Fig S4C and A). This mislocalization entailed a drastic down-regulation of the A, B1 and E1 subunit levels relative to WT as quantitatively determined in Western blots of kidney lysates from n = 5 WT and *n* = 5 *Atp6v0a4*^−/−^ mice (A subunit WT: 1.00 ± 0.05, KO: 0.33 ± 0.07, *p* < 0.0001; B1 subunit WT: 1.00 ± 0.08, KO: 0.17 ± 0.02, *p* < 0.0001; E1 subunit WT: 1.000 ± 0.09, KO: 0.26 ± 0.07, *p* < 0.0001; Fig 2G). This suggests that the absence of the a4 subunit interferes with the expression or the stability of other V-ATPase subunits. This was further corroborated by immunogold labelling of the A subunit. It localized to the apical plasma membrane and intracellular vesicles of type A-ICs of WT mice, but was almost absent in *Atp6v0a4*^−/−^ mice (Fig 2J and K).

**Figure 3:**
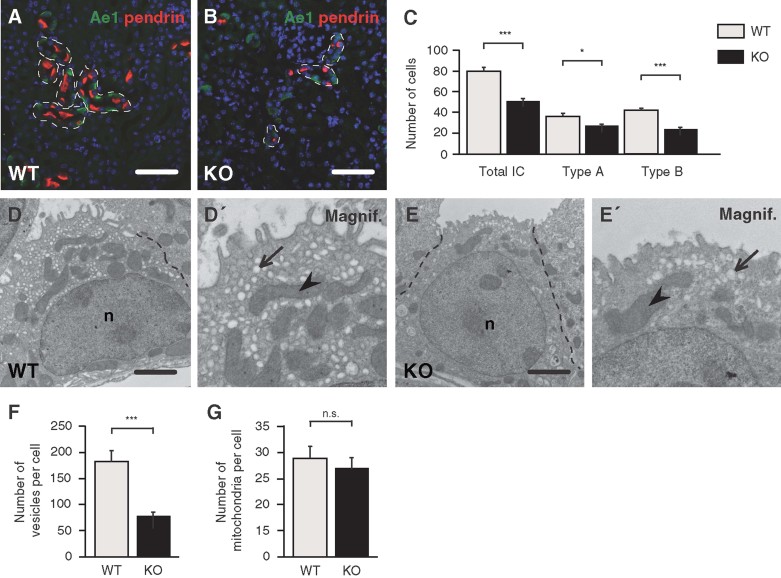
Characterization of ICs in a4 KO mice **A,B.** Immunofluorescence for Ae1 (green) and pendrin (red) in WT and *Atp6v0a4*^−/−^ mice. Scale bars: 100 µm. The basolateral borders of collecting ducts are indicated by dashed lines.**C.** Quantification of IC phenotype in wildtype and *Atp6v0a4*^−/−^ mice reveals a reduction of total ICs, type A- and type B-ICs in a4 KO mice (***p < 0.001; *p < 0.05).**D,E.** Electron micrographs of ICs show a reduced number of vesicles (arrows) but not of mitochondria (arrowheads) in the a4 KO compared to WT. Scale bars: 2 µm, *n*: nucleus. The lateral cell borders are indicated by a dashed line.**D′,E′.** Magnification of the respective overview electron micrograph in **D** and **E**.**F,G.** Quantification of intracellular vesicles and mitochondria in ICs from WT and *Atp6v0a4*^−/−^ mice reveals a reduced number of intracellular vesicles (***p < 0.0001), but no significant difference for mitochondria.

In response to metabolic acidosis, the total number of acid secreting A-ICs is expected to increase while a parallel decrease in base secreting B-ICs occurs, the total number of ICs being unchanged (Schwartz & Al-Awqati, 1986; Schwartz et al, 1985). Although we observed a dramatic reduction in the number of pendrin-positive cells, reflecting the expected decrease in type B-ICs in response to acidosis, there was no compensatory increase, but rather a decrease in the number of Ae1-positive ICs (Fig 3A–C). In Western blots of kidney lysates from *n* = 5 *Atp6v0a4*^−/−^ mice pendrin was significantly reduced compared to WT (WT: 1.00 ± 0.07, KO: 0.08 ± 0.01; *p* < 0.0001; Fig 2G). Moreover, electron microscopy studies of ICs (Fig 3D–E) from two independent mice per genotype showed significantly decreased numbers of intracellular vesicles in *Atp6v0a4*^−/−^ mice (75 ± 5 vesicles per cell, *n* = 15) compared to WT (187 ± 20 vesicles per cell, *n* = 11; *p* < 0.0001; Fig 3F and Supporting Information Fig S5A–B) suggesting that the remaining ICs were in a compromised functional state. The number of mitochondria per cell, however, did not differ between genotypes (WT: 29 ± 2 mitochondria, *n* = 11; KO: 27 ± 2 mitochondria, *n* = 13, n.s.; Fig 3G).

Because the metabolic acidosis of *Atp6v0a4*^−/−^ mice was surprisingly severe compared to that observed in B1 KO mice (Finberg et al, 2005), we reanalysed the clinical data that were available for 44 patients with recessive dRTA associated with *ATP6V0A4* mutations and 45 patients associated with mutations in *ATP6V1B1* (Table 2), some of which had been published previously (Vargas-Poussou et al, 2006). Importantly, the age at diagnosis (0.35 ± 0.14 years compared to 1.55 ± 0.58 years; *p* = 0.0001), the blood pH (7.18 compared to 7.28; *p* = 0.002) and the pCO_2_ (12.0 *vs.* 13.8 mmol/L; *p* = 0.033) were lower in patients with a defect of the a4 subunit. In urine of healthy subjects, pH varies between pH 8.0 and 5.5 depending on the net acid or alkali load content of the food. Under the stimulus of metabolic acidosis, a urine pH above pH 5.5 is considered pathological (Morris & McSherry, 1972). Hence, the urine pH of 7.47 ± 0.11 in patients with *ATP6V0A4* mutations and of 7.25 ± 0.078 in patients with mutations in *ATP6V1B1* suggests a distal acidification defect in both groups. Furthermore, episodes of dehydration as an initial manifestation of disease were more frequent in patients with mutations in *ATP6V0A4* (14/38 *vs*. 6/41; *p* = 0.024). Obviously, as in mice, the phenotype of patients carrying mutations in *ATP6V0A4* was more severe as compared to patients related to *ATP6V1B1* mutations.

**Table 2:** Patients carrying mutations in the a4 subunit are more severely affected in terms of age at diagnosis, severity of acidosis and dehydration episodes than B1 subunit-related patients

	*ATP6V0A4* (*n* = 44)	*ATP6V1B1* (*n* = 45)	*p*-value
Age at diagnosis (years)	0.35 ± 0.136^2^	1.55 ± 0.582^1^	***
More frequent first manifestations			
Failure to thrive	21/38	27/41	n.s.
Dehydration	14/38	6/41	*
Vomiting	8/38	9/41	n.s.
Polyuria	3/38	6/38	n.s.
Sensorineural hearing loss (SNHL)	17/44	33/45	**
Age at diagnosis of SNHL (years)	5.3 ± 1.325^3^	3.4 ± 0.799^6^	n.s.
Plasma/Blood			
pH	7.15 ± 0.023^21^	7.24 ± 0.027^11^	***
Na^+^ (133–146 mmol/L)	137.7 ± 0.939^26^	139.0 ± 0.719^20^	n.d.
K^+^ (3.5–5 mmol/L)	2.99 ± 0.109^10^	3.00 ± 0.111^8^	n.s.
Cl^−^ (90–117 mmol/L)	113.0 ± 1.505^16^	113.7 ± 1.323^16^	n.s.
CO_2_ (18–25 mmol/L)	11.48 ± 0.626^6^	13.49 ± 0.495^3^	*
Ca^2+^ (2.20–2.70 mmol/ L)	2.89 ± 0.098^13^	2.53 ± 0.056^13^	*
P_i_ (1–4 y 1.29–2.20 mmol/L)	1.63 ± 0.119^34^	1.59 ± 0.093^34^	n.d.
Urine			
pH during acidosis	7.47 ± 0.110^18^	7.25 ± 0.078^9^	n.s.
UNa^+^ (mmol/L)	28 ± 3.323^30^	43.8 ± 5.959^30^	n.d.
UK^+^ (mmol/L)	25 ± 2.628^26^	32 ± 3.499^29^	n.d.
UCl^−^ (mmol/L)	47.5 ± 16.540^36^	29.7 ± 5.885^37^	n.d.
UCa^2+^/UCrea (mmol/mmoL)	2.64 ± 0.305^23^	1.36 ± 0.259^29^	n.d.

Values are expressed as mean ± SE. Superscript values correspond to the number of missing data.

The *p* values are given for the comparison of the two groups by the Mann–Whitney test with (n.s., not significant; *p < 0.05, **p < 0.01, ***p < 0.001); n.d., not determined (more than 50% of missing data).

### Atp6v0a4 deficiency impairs proximal tubule function

**Figure 4:**
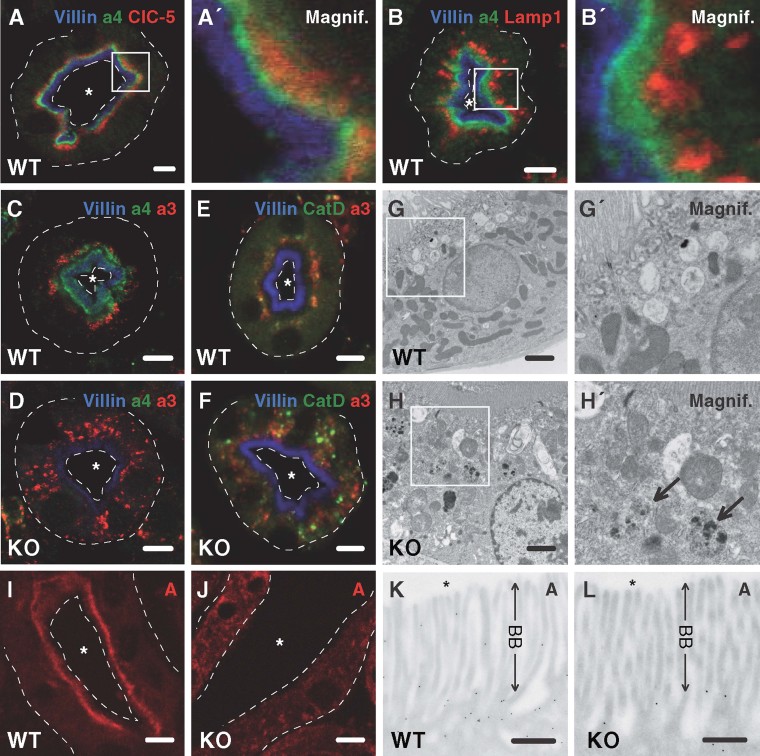
Accumulation of lysosomal material in proximal tubules of a4 KO mice For clarity, the basolateral and apical borders of the tubular epithelium are indicated by dotted lines and the lumina are marked with an asterisk in immunofluorescence images. **A-C.** The a4 subunit (green) partially co-localizes with the endosomal marker ClC-5, but not with the lysosomal marker Lamp1 (red) or the lysosomal a3 V-ATPase subunit (red). The brush border membrane is stained with Villin (blue). Scale bars: 5 µm.**A′,B′.** Magnification of the respective area in **A** and **B**.**D.** The section of the a4 KO mouse kidney shows more, and more broadly distributed lysosomal vesicles as demonstrated by a3-positive (red) intracellular vesicles. Scale bar: 5 µm.**E,F.** Altered lysosomal structures in an a4 KO mouse kidney section as compared to WT as characterized by co-staining with Cathepsin D (green) and a3 (red). Brush border marked by Villin (blue). Scale bars: 5 µm.**G,H.** Electron micrographs of proximal tubules show considerable amounts of vesicular storage material (arrows) in the a4 KO compared to WT. Scale bars: 2 µm.**G′,H′.** Magnification of the respective area of the overview electron micrograph in **G** and **H**. The insets in all cases (electron micrographs) are 2.4× higher mags than the lower mags and this means that the scale bar showing 2 µm on the lower mag figures represents 0.8 microns in the inset.**I,J.** The plasma membrane association of the A subunit (red) is lost in the a4 KO mouse. Scale bars: 5 µm.**K,L.** Immunogold staining for the A subunit reveals less V-ATPase below the brush border (BB) membrane in the a4 KO, but no morphological changes of microvilli. Scale bars: 500 nm.

**Figure 5:**
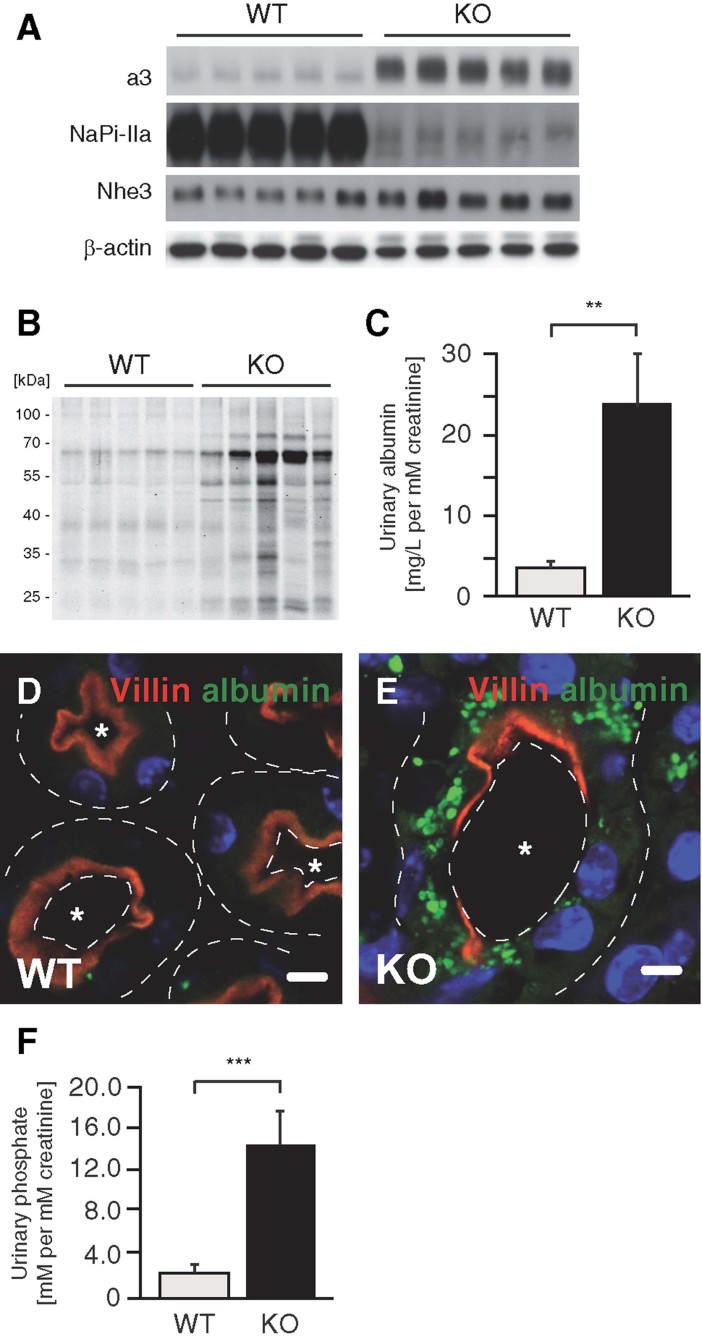
Proximal tubule dysfunction in a4 KO mice For clarity the basolateral and apical borders of the tubular epithelium are indicated by dotted lines and the lumina are marked with an asterisk in immunofluorescence images. **A.** Western Blot analysis of kidney lysates of five individual mice per genotype shows a significant increase of the a3 subunit and Nhe3 expression levels in kidney lysates of a4 KO mice. Expression of NaPi-IIa is strongly decreased in whole kidney protein lysates of a4 KO mice. β-actin serves as a loading control.**B.** Visualization of urinary proteins by SDS–polyacrylamide gel electrophoresis and Coomassie staining. Loading volume was adjusted to creatinine levels.**C.** Albumin ELISA verifies proteinuria in a4 KO spot urine samples (**p < 0.01).**D,E.** Accumulation of intracellular albumin (green) deposits in epithelial cells of the proximal tubule of the a4 KO. Villin (red), DAPI (blue). Scale bars: 5 µm.**F.** Urinary phosphate levels are increased in a4 KO mice compared to WT mice (***p < 0.01).

Proximal tubule cells have an elaborate endocytic machinery to recover proteins from the primary filtrate, which are subsequently degraded within lysosomes, a process that critically depends on the activity of the V-ATPase (Marshansky et al, 2002; Marshansky & Futai, 2008). In these cells, a4 labelling was observed in a sub-apical compartment close to the membrane (Fig 4A and B and Supporting Information Fig S1A–C). This compartment overlapped only partially with ClC-5, a Cl^−^/H^+^antiporter, which localizes to endosomes (Günther et al, 1998; Fig 4A). In contrast, Lamp1 or the Atp6v0a3 (a3) subunit of the V-ATPase, which localize to the lysosomal compartment (Chen et al, 1985; Toyomura et al, 2003), did not co-localize with the a4 subunit (Fig 4B and C). Surprisingly, *Atp6v0a4*^−/−^ mice showed increased numbers of a3-, cathepsin D (CatD)- and ClC-7-positive vesicles in proximal tubule cells (Fig 4C–F and Supporting Information Fig S6A and B). In electron micrographs of proximal tubule cells, we observed considerable amounts of vesicular storage material in *Atp6v0a4*^−/−^ mice compared to WT mice (Fig 4G and H; for an overview of whole proximal tubule sections see Supporting Information Fig S7A and B). An alteration of the lysosomal compartment was further corroborated by a robust up-regulation of the lysosomal V-ATPase a3 subunit in Western blot studies of kidney protein lysates (Fig 5A). To exclude systemic non-specific effects of the severe metabolic acidosis on the subcellular localization of lysosomes (Heuser, 1989), we also analysed the lysosomal compartment in *Atp6v0a4*^−/−^ enterocytes of the small intestine, which are organized similarly to proximal tubule cells but lack the a4 subunit even in WT. We did not find any differences between WT and *Atp6v0a4*^−/−^ enterocytes (Supporting Information Fig S8A and B). Similarly arguing against a non-specific effect of acidosis on lysosomes, Western blots indicated that the abundance of the lysosomal V-ATPase a3 subunit was markedly increased even in heterozygous mice compared to controls (Supporting Information Fig S8C).

As shown in ICs, the immunoreactivity for the A subunit of the V-ATPase was changed in proximal tubule cells in *Atp6v0a4*^−/−^ mice as well (Fig 4I and J). Together with its down-regulation (as quantified by Western analysis of kidney protein lysates) this indicated that the expression and/or assembly of the V-ATPase complex were severely perturbed in the absence of the a4 subunit. This was further corroborated by immunogold labelling for the A subunit in proximal tubule cells (Fig 4K and L). Whereas the immunogold labelling for the A subunit was drastically decreased, this analysis did not reveal any obvious structural alterations in the brush border membranes of *Atp6v0a4*^−/−^ mice (Fig 4K and L).

To assess consequences of the disruption of the a4 subunit on endocytosis, we analysed urinary proteins by SDS–polyacrylamide gel electrophoresis and subsequent Coomassie staining. Supporting an endocytosis defect, urinary protein levels and in particular albumin (as quantified by ELISA) were increased in urine samples of *Atp6v0a4*^−/−^ (24.13 ± 6.02 mg/L per mM creatinine, *n* = 5) compared to WT mice (3.92 ± 0.66 mg/L per mM creatinine, *n* = 7, *p* < 0.01) (Fig 5B–C). However, endocytosis of albumin was obviously partially preserved, as albumin-positive intracellular vesicles could be detected in proximal tubules of *Atp6v0a4*^−/−^ mice (Fig 5E). The fact that such an accumulation of intracellular albumin was not observed in WT proximal tubules (Fig 5D) strongly suggested a defect of the degradative pathway in the absence of the a4 subunit. A drastic reduction of the apical phosphate transporter NaPi-IIa (Slc34a1, Npt2a) in the brush border of proximal tubule cells in the kidney was another indication for proximal tubular dysfunction (Fig 5A). NaPi-IIa has been shown to traffic between the endocytic compartment and the plasma membrane (Beck et al, 1998; Shenolikar et al, 2002). The decrease of NaPi-IIa was specific, since the Na^+^/H^+^ exchanger Nhe3, another brush border membrane protein of proximal tubule cells, was moderately up-regulated (Fig 5A) as can be expected for acidotic mice (Eladari et al, 2002). In agreement with reduced NaPi-IIa expression, urinary phosphate levels were significantly increased in *Atp6v0a4*^−/−^ mice (Fig 5F).

Taken together, our data support the concept that disruption of the a4 subunit interferes with proximal tubule function and might thus contribute to the phenotypic spectrum of patients carrying mutations in *ATP6V0A4*.

### Disruption of Atp6v0a4 results in an enlargement of the endolymphatic compartment and profound deafness

**Figure 6:**
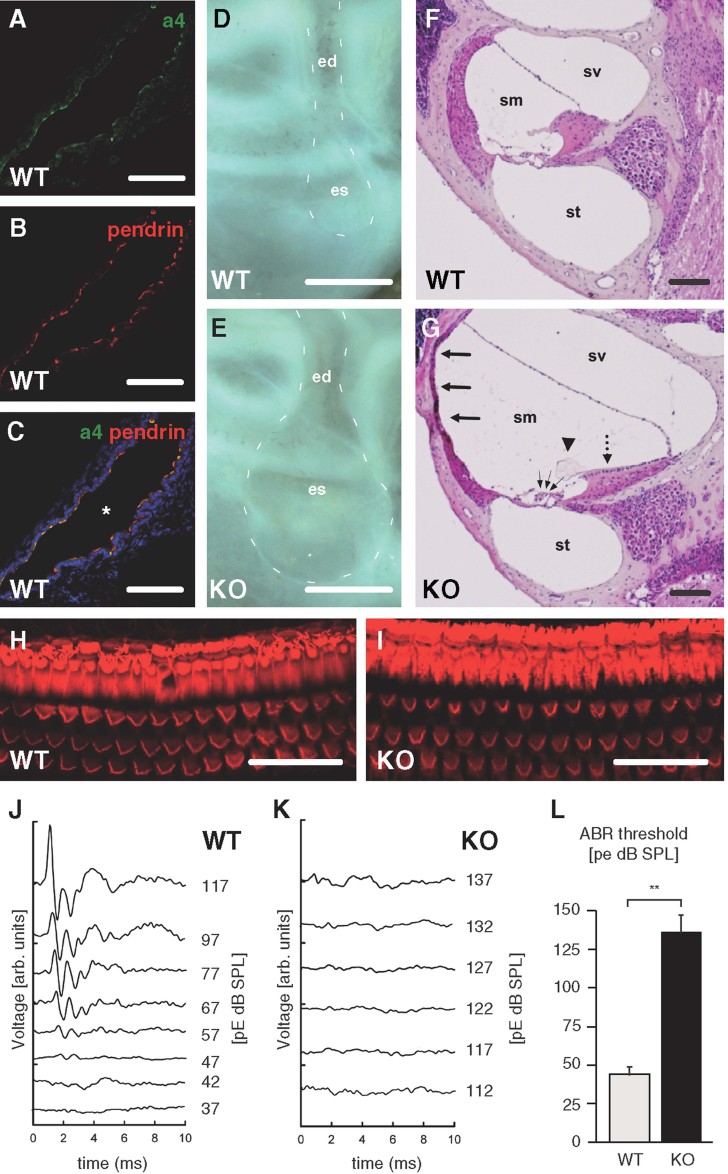
Enlargement of the endolymph compartment in a4 KO mice **A-C.** a4 (green) and pendrin (red) co-localize in epithelial cells of the endolymphatic sac at postnatal day (P) 1. Both proteins localize to the apical pole of endolymphatic sac (es) epithelial cells. Nuclei are stained with DAPI (blue). The overlay is shown in (**C**). Scale bars: 100 µm. The lumen is highlighted by an asterisk.**D,E.** The endolymphatic sac (es) and duct (ed), indicated by dotted lines are dilated at P1 in the a4 KO. Scale bars: 150 µm.**F,G.** At P18, the a4 KO displays an enlargement of the scala media (sm), compression of spiral ligament fibrocytes (three large arrows), flattening of the spiral limbus (dotted arrow) and hypertrophy of the tectorial membrane (arrowhead), whereas outer hair cells are preserved (three small arrows). sv, scala vestibuli; st, scala tympani. Scale bars: 100 µm.**H,I.** Phalloidin-stained inner and outer hair cell bundles (lower three rows) confirm the preservation of hair cells at P21. Scale bars: 20 µm.**J-L.** Auditory brainstem responses to acoustic clicks are absent in a4 KO mice (**p = 0.01).

The V-ATPase is highly expressed in several cell types in the inner ear (Stanković et al, 1997) and dRTA caused by a4 mutations in humans can be accompanied by hearing impairment (Stover et al, 2002; Vargas-Poussou et al, 2006). Therefore, we analysed the expression of the a4 subunit in the inner ear. At postnatal day P1, a4 was confined to the luminal side of epithelial cells of the endolymphatic sac, where it co-localized with pendrin (Fig 6A–C). In E15 and P1 mice lacking the a4 subunit, the endolymphatic duct as well as the endolymphatic sac were enlarged (Fig 6D–E). An enlargement of the endolymphatic space was also evident in HE-stained cross sections of the inner ear of P18 *Atp6v0a4*^−/−^ mice. In particular, the cochlear duct, the scala media and the scala vestibularis were massively dilated, whereas inner and outer hair cells as well as neurons of the spiral ganglion appeared to be preserved. Fibrocytes of the spiral ligament as well as the stria vascularis were compressed and the spiral limbus flattened (Fig 6F and G), findings that are reminiscent of inner ear malformations in pendrin KO mice and in deaf individuals carrying mutations in the gene encoding Pendrin (Everett et al, 2001). At P21, we did not detect a loss of inner and outer hair cells in phalloidin-stained whole mount specimens of the organ of Corti (Fig 6H and I), although the auditory brainstem responses to acoustic clicks (Fig 6J and L) showed that *Atp6v0a4*^−/−^ mice were profoundly deaf at that age (WT: 43.67 ± 4.29 pe dB SPL (*n* = 6), KO: 133.25 ± 11.93 pe dB SPL (*n* = 5); *p* = 0.01).

## DISCUSSION

Here, we show that mice with a targeted disruption of the a4 subunit displayed proximal tubule dysfunction and a severe defect of renal acid secretion. The co-localization of the a4 subunit with the E1 or A subunit of the V-ATPase suggested that it is directly involved in proton secretion by type A ICs. As a correlate of impaired acid secretion, the loss of the a4 subunit also entailed drastic morphological changes of type A-ICs with a marked reduction of specialized V-ATPase transporting vesicles.

The severity of metabolic acidosis upon a4 subunit disruption contrasted markedly with the very mild effects observed in mice with a disruption of the B1 subunit (Karet et al, 1999) which have been attributed to a partial compensation by the incorporation of the alternative B2 subunit isoform into the V-ATPase holoenzyme (Paunescu et al, 2007). On the contrary, the drastic decrease of the V-ATPase E1 or A subunits in *Atp6v0a4*^−/−^ mice suggests that the expression and/or assembly and/or targeting of the V-ATPase is severely impaired in the absence of a4. Indeed, signals necessary to target the V-ATPase to different cellular destinations were localized to the amino-terminal domain of the a subunit (Kawasaki-Nishi et al, 2001). Moreover, our findings are in agreement with the observation that the knockdown of the a4 subunit in a breast cancer cell line could not be functionally compensated by an up-regulation of another V-ATPase subunit (Hinton et al, 2009).

One has to consider that loss of the a4 subunit may also affect proximal acid secretion, in contrast to the B1 subunit, which is not expressed in the proximal tubule (Nelson et al, 1992). Even though the V-ATPase is present in the apical membrane of proximal tubule cells, its role in urine acidification is still under debate. Physiological studies initially concluded that the V-ATPase may mediate up to 40% of proton secretion in this segment (Chan & Giebisch, 1981) and that the V-ATPase is up-regulated in response to metabolic acidosis (Chambrey et al, 1994), but subsequent studies emphasized the critical role of Nhe3 in this process. However, in the absence of suitable mouse models with a proximal tubule-specific deletion of the pump, the contribution of each protein to proximal urine acidification remains unknown. Reanalysis of clinical data revealed that patients carrying mutations in the a4 subunit were more severely affected as well in terms of age at diagnosis and severity of acidosis as compared to patients with mutations of the B1 subunit.

The defect of the V-type ATPase might also impair the function of type B-ICs, which secrete bicarbonate apically via pendrin and recover H^+^ basolaterally via the V-ATPase. As secretion of bicarbonate would be counterproductive in metabolic acidosis, the stark down-regulation of pendrin in *Atp6v0a4*^−/−^ mice may be a consequence of metabolic acidosis. Recent evidence points to an involvement of pendrin together with the Na^+^-coupled anion-exchanger NDCBE in the reabsorption of NaCl (Leviel et al, 2010). Hence, the secondary down-regulation of pendrin may result in a loss of NaCl, a symptom, which often complicates dRTA in patients (Sebastian et al, 1971). Our data showing that patients with *ATP6V0A4* mutations were diagnosed earlier, had a more severe metabolic acidosis and a higher frequency of dehydration episodes are consistent with the severity of the phenotype observed in *Atp6v0a4*^−/−^ mice and support a role of the a4 subunit in NaCl reabsorption. However, because of the very early lethality of a4 disruption in mice it was not possible to carefully dissect the impact of the knockout on sodium balance and on vascular volume and blood pressure regulation in this work. This will require additional studies upon IC-specific deletion of the a4 subunit.

The proteinuria, phosphaturia and lysosomal defects in *Atp6v0a4*^−/−^ mice show that the a4 subunit is also crucial for normal proximal tubule function. In support of the clinical relevance of these findings, we identified a patient with a4-related dRTA who had initially been misdiagnosed with Dent's disease because of pronounced proximal tubular proteinuria (β-2 microglobulinuria: 17.11 mg/mmol of creatinine) and global aminoaciduria. The partial overlap with the 2Cl^−^/H^+^ antiporter ClC-5 and the lack of co-localization with the lysosomal 2Cl^−^/H^+^antiporter ClC-7 and Lamp1 suggest that the a4 subunit does not reside in lysosomes and late endosomes, but in earlier endocytic compartments. The abnormal localization of the A subunit of the V-type ATPase furthermore suggests that the assembly of the V-type ATPase is critically disturbed in the absence of the a4 subunit. This is likely to have various secondary effects on both upstream and downstream compartments of the endocytic apparatus. The GTPase Arf6 and its cognate GDP/GTP exchange factor ARNO, which directly interact with different subunits of the V-ATPase, are a particularly striking example. Their recruitment from the cytosol to endosomal membranes is driven by V-ATPase-dependent intra-endosomal acidification (Hurtado-Lorenzo et al, 2006). Moreover, disturbing this interaction resulted in reduced endocytosis and prevented the delivery of endocytosed proteins from early to late endosomes, thus causing an accumulation of cargo in early endosomes and, ultimately, inhibition of endocytosis (Hurtado-Lorenzo et al, 2006). An acidification-independent role of the V0 domain in membrane fusion (Bayer et al, 2003; Peters et al, 2001) may also contribute to the defect of the degradative pathway. In *Drosophila melanogaster*, loss-of-function mutations in a neuron-specific isoform of the a-subunit blocked synaptic vesicle fusion without affecting synaptic vesicle acidification (Hiesinger et al, 2005).

Western blot analysis demonstrated a drastic down-regulation of NaPi-IIa in the proximal tubule, although metabolic acidosis as such may increase NaPi-IIa protein levels (Murer & Biber, 2010). Apart from a trafficking defect, the down-regulation of NaPi-IIa may also result from abnormally high levels of urinary parathyroid hormone (PTH). As shown for ClC-5 KO mice (Piwon et al, 2000), impaired proximal tubular endocytosis can increase luminal levels of PTH, which in turn stimulates the internalization and degradation of NaPi-IIa. Surprisingly, and unlike ClC-5 KO mice (Piwon et al, 2000; Wartosch et al, 2009), *Atp6v0a4*^−/−^ mice displayed an enlargement of the lysosomal compartment as demonstrated by an increased number of Lamp1-, ClC-7- or a3-positive vesicles and an accumulation of undegraded material in proximal tubule cells, findings that superficially resembled the alterations observed upon disruption of the lysosomal ClC-7 transporter (Wartosch et al, 2009). As the a4 subunit was not expressed in the lysosomal compartment, it is possible that the accumulation is caused by an abnormal lysosomal composition due to a sorting defect (Nielsen et al, 2007).

Hearing impairment in patients with dRTA related to either *ATP6V0A4* or *ATP6V1B1* mutations is variable both in terms of onset and severity. As mice with a disruption of the B1 subunit had normal hearing (Karet et al, 1999), we were surprised that mice lacking the a4 subunit were already profoundly deaf in their second week of life. At this time point, inner and outer hair cells as well as the neurons within the spiral ganglion appeared to be preserved, whereas the scala media was significantly enlarged. These latter findings resembled alterations in mice lacking the anion transporter pendrin. Mutations in Pendrin are a common cause of deafness and probably the most common cause for hereditary enlargement of the vestibular aqueduct, a frequent malformation in children with sensorineural hearing loss (Phelps et al, 1998). The formation of the lumen of the inner ear depends critically on the secretion of fluid into the vestibular labyrinth, which is reabsorbed in the endolymphatic sac. Importantly, mice devoid of pendrin during a period between E16.5 and P2 displayed abnormally acidic endolymph, loss of the endocochlear potential and were profoundly deaf later on (Choi et al, 2011). As the a4 subunit co-localized with pendrin in the apical membrane of epithelial cells of the endolymphatic sac in this time window, this suggests that the V-ATPase is involved in fluid absorption and ion homeostasis in the developing ear and may serve to counterbalance bicarbonate secretion by pendrin. Expression in epithelial cells of the endolymphatic sac of the developing mouse ear has also been reported for the B1 and other V-ATPase subunits (Karet et al, 1999; Stanković et al, 1997) and enlargement of the vestibular aqueduct has been observed in patients with mutations in either *ATP6V1B1* or *ATP6V0A4* (Andreucci et al, 2009). Thus, deafness in a4 KO mice is most likely owed to an abnormal composition of the endolymph and possibly to a secondary alteration of the endocochlear potential. In barttin KO mice, *e.g.* the decrease of the endocochlear potential was sufficient to cause profound deafness although inner and outer hair cells were initially preserved (Rickheit et al, 2008).

Taken together, we show that the a4 subunit is not only essential for endolymph homeostasis and distal tubular acidification, but also plays a critical role in the proximal tubule. These findings imply that the complex phenotype of dRTA patients related to *ATP6V0A4* mutations may in part be a consequence of an impairment of proximal tubular function.

## MATERIALS AND METHODS

All animal experiments were approved by the Thüringer Landesamt für Lebensmittelsicherheit und Verbraucherschutz (TLLV) in Germany. For genetic studies, written informed consent was obtained from the patients or their parents according to French legislation. Clinical data at diagnosis were obtained as a part of the aspects necessary for orientation of genetic studies and analysed retrospectively.

### Generation of *Atp6v0a4* deficient mice

A clone isolated from a 129/SvJ mouse genomic λ library (Stratagene, now Agilent, Santa Clara, CA, USA) was used to construct the targeting vector (Rust et al, 2007). An approximately 11 kb *Sal*I/*Spe*I fragment including exons 10–13 of the *Atp6v0a4* gene was cloned into the pKO-V901 plasmid (Lexicon Genetics, The Woodlands, TX, USA) with a phosphoglycerate kinase (pgk) promoter-driven diphtheria toxin A cassette. A pgk promoter-driven neomycin resistance cassette flanked by frt sites and an additional loxP site was inserted into the *Age*I site of intron 11. A second loxP site and an additional *Bam*HI site were inserted into the *Mlu*I site in intron 10. The construct was linearized with *Not*I and electroporated into R1 mouse embryonic stem (ES) cells. Neomycin-resistant clones were analysed by Southern blot using *Bam*HI and an external approximately 400-bp probe (NC_000072 *Mus musculus* chromosome 6, 38009912-38010276). Two correctly targeted ES cell clones were injected into C57BL/6 blastocysts to generate chimeras. Chimeric mice were mated to a cre-Deleter mouse strain to remove exon 11 and the selection cassette (Schwenk et al, 1995). Studies were performed in a mixed 129Sv/C57BL/6 background in the F4 and F5 generation. Genotypes were determined by PCR of tail biopsy DNA. For PCR genotyping, the forward primers F1 (5′-caaggcctgagatcctgagtt-3′), F2 (5′-ggatgagttagcaaagtggctg-3′) and the reverse primer R1 (5′-ctcaaccaagtcttccctagg-3′) were used in a single PCR mix. The primer pair F1/R1 amplified a 378-bp WT allele, and the primer pair F2/R1 a 275-bp KO allele.

**The paper explained**

PROBLEM:

Mutations in the gene encoding the a4 and the B1 subunit of the vacuolar-type H^+^-ATPase lead to dRTA and hearing loss of variable degree. Apart from metabolic acidosis, a significant number of dRTA patients also develop nephrocalcinosis, polyuria, dehydration and hypokalemia. Thus, dRTA patients share symptoms with patients suffering from Dent's disease, which is a disorder of the proximal tubule caused by abnormal endosomal acidification. As the a4 subunit, in contrast to the B1 subunit, is also expressed in endosomes of the proximal tubule, we hypothesized that a defect of the a4 subunit might have consequences for proximal tubule function and thus contribute to the complex phenotype of some of the patients with dRTA.

RESULTS:

To study the pathogenesis of a4-related dRTA, we disrupted the a4 subunit encoding gene *Atp6v0a4* in mice. a4-deficient mice developed severe metabolic acidosis because of a distal acid secretion defect, which is in sharp contrast to the mild phenotype of mice devoid of the B1 subunit (Finberg et al, 2005). Indeed, re-analysis of clinical data revealed that a4-related dRTA patients were also more severely affected as compared to patients with mutations of the B1 subunit.

In addition to the distal acid secretion defect, a4-deficient mice displayed phosphaturia, proteinuria and an impaired degradative pathway of proximal tubule cells with accumulation of lysosomal material. These findings indicate a severe dysfunction of the proximal tubule upon disruption of the a4 subunit, a ‘hidden’ pathology that we also identified in a patient suffering from a4-related dRTA initially misdiagnosed with Dent's disease.

In the inner ear, the a4 subunit co-localized with the anion-exchanger pendrin at the apical side of epithelial cells lining the endolymphatic sac. Moreover, mice devoid of the a4 subunit were profoundly deaf and displayed enlarged endolymph compartments, mirroring the alterations in mice with a targeted disruption of the anion-exchanger pendrin.

IMPACT:

Our study provides unique and novel insights into the mechanisms of metabolic acidosis and hearing loss caused by *ATP6V0A4* mutations. It also reveals a previously unrecognized role for the a4 subunit in endocytic trafficking in epithelial cells of the proximal tubule. Thus, our findings require a revision of the current dogma that kidney disease in dRTA arises only from defects in the distal tubule.

### Northern blot

The *Atp6v0a4* 3′ UTR probe was cloned by PCR using the forward primer 5′-tcactcttgtctctgacat-3′ and reverse primer 5′-aaagtcaaggacatcctt-3′. Northern blot analysis was performed as described previously (Hübner et al, 2001).

### Renal function

3–4-week-old *Atp6v0a4*^+/+^ (WT, *n* = 5), *Atp6v0a4*^+/−^ (Het, *n* = 6) and *Atp6v0a4*^−/−^ (KO, *n* = 5) mice were explored for their renal function. Spot urine was collected and pH was measured with a pH microelectrode (Inlab Ultra-micro pH, Mettler, Viroflay, France). Urinary ammonium and creatinine concentrations were measured as described previously (Chambrey et al, 2005). Blood pH and pCO_2_ were measured with the ABL77 pH/blood–gas analyser (Radiometer, Copenhagen, Denmark). Blood bicarbonate concentration was calculated from pH and pCO_2_ using the Henderson–Hasselbalch equation. For acid loading *n* = 9 WT and *n* = 7 Het mice obtained water supplemented with 0.28M NH_4_Cl as drinking water.

### Immunogold staining and electron microscopy

For immunogold staining, small pieces of WT and a4 KO kidneys were dehydrated through a graded series of ethanol to 100% ethanol and then embedded in LR White resin (Electron Microscopy Sciences, Fort Washington, PA, USA). Thin sections were incubated on drops of primary anti-V-ATPase (A-subunit, 1:200) antibody for 2 h. After rinsing with PBS, the grids were incubated on drops of goat anti-rabbit IgG coupled to 15 nm gold particles (Ted Pella, Redding, CA, USA) for 1 h. Following several rinses with distilled water, the grids were stained with 2% uranyl acetate for 10 min, rinsed in distilled water, and dried. Sections were examined in a JEM-1011 transmission electron microscope (JEOL Ltd., Tokyo, Japan). For conventional electron microscopy, some kidneys were fixed in 2.5% glutaraldehyde in 0.1 M cacodylate buffer, pH 7.4. They were post fixed for 1 h in 2% osmium tetroxide, stained en bloc with uranyl acetate, dehydrated in graded ethanol and embedded in Epon (Electron Microscopy Sciences, Ft. Washington, PA, USA). Thin sections were stained with uranyl acetate and lead citrate prior to examination.

### Immunofluorescence

In general, all immunostainings were performed on sections of at least three independent mice per genotype if not indicated otherwise and only representative findings are presented in the figures. Kidneys were fixed by *in vivo* perfusion of 4% paraformaldehyde in phosphate buffered saline (1× PBS). Antigens were retrieved prior to the labelling procedure by heating the sections in 0.01 M Tris, 0.1 M EDTA buffer for 5 min at 96°C or 1% SDS in 1× PBS. Sections were stained as described (Boettger et al, 2002) and analysed with a confocal microscope (Leica TCS SP5, Wetzlar, Germany or Zeiss LSM 510 META, Göttingen, Germany).

Inner ears were removed, post-fixed for 2 h in 4% PFA in 1× PBS and decalcified by 10% EDTA/1× PBS at 37°C overnight (decalcification of inner ears was done in mice > P2 only). For immunofluorescence 8 µm cryosections were cut and processed as described above. For phalloidin staining, basilar membranes of fixed inner ears were removed and stained with Alexa-555-conjugated phalloidin in 1× PBS/TX-100 for 20 min. Haematoxylin and eosin (HE) histology was performed on 8 µm paraffin sections following standard protocols.

### Intercalated cell type quantification

Kidney sections from *Atp6v0a4*^+/+^ and *Atp6v0a4*^−/−^ mice were co-stained with either Ae1 or Atp6v1e1 (E subunit of the V1 domain) or pendrin and E1, respectively. Randomly photographed cortex areas (*n* = 12 fields) were analysed for co-expressing cells by blinded experimenters and the total number of ICs was calculated with standard error as the sum of both sub-populations. For the quantification of intracellular vesicles and mitochondria in ICs at least 10 cross-sections from ICs of two different mice per genotype were evaluated.

### Western blot analysis

Isolated kidneys were homogenized in isolation buffer (250 mM sucrose, 20 mM Tris–Hepes, pH 7.4) containing a protease inhibitor cocktail (Roche Diagnostics, Risch, Switzerland). After removal of cell debris the kidney lysates were centrifuged at 17,000 *g* at 4°C for 30 min and the membrane proteins resuspended in isolation buffer. Protein concentration was determined using the Bradford protein assay (Bio-Rad Laboratories, Hercules, CA, USA). For Western Blotting 10–60 µg protein was solubilized in SDS-loading buffer (62.5 mM Tris–HCl, pH 6.8, 2% SDS, 100 mM dithiotreitol, 10% glycerol and bromophenol blue) and incubated at room temperature for 30 min. Proteins were separated on reducing 7.5% SDS–polyacrylamide gels and loading controls were performed as described (Quentin et al, 2004). For Western blot of spot urine, urine samples were normalized to urine creatinine values. Briefly, urine resuspended in SDS loading buffer was resolved on a 7.5% polyacrylamide gel in Tris–glycine buffer and in gel protein staining was carried out with Coomassie Blue (Euromedex, Souffelweyersheim, France).

### Urine albumin measurements

To detect mouse albumin spot urine samples from *n* = 7 WT and *n* = 5 KO mice were analysed by an enzyme-linked immunosorbent assay (ELISA) against mouse albumin (AssayMax Mouse albumin ELISA Kit, AssayPro, St. Charles, MO, USA) according to the instructions of the manufacturer. The measurement was repeated twice.

### Antibodies

*Primary antibodies*: guinea pig anti-mouse Ae1 (Stehberger et al, 2007) (1:5000), rabbit/guinea pig anti-mouse Atp6v0a3 (Lange et al, 2006) (1:100), rabbit anti-human Atp6v0a4 (Stehberger et al, 2003; 1:500), rabbit anti-mouse Atp6v1b1 (Vallet et al, 2006; 1:30,000), chicken anti-Atp6v1e1 (1:500; Breton et al, 2000), rabbit anti-ClC-5 (Günther et al, 1998; PEP5E, 1:100), rabbit anti-mouse ClC-7 (Kornak et al, 2001; 7N4B, 1:100), rat anti-mouse Lamp1 (BD Biosciences, 1:250), rabbit anti-rat Na^+^/P_i_ cotransporter 2a (NaPi-IIa, gift of J. Biber, Zürich, Switzerland, 1:1000), rabbit anti-rat Nhe3 (Kim et al, 1999a; 1:5000), guinea pig anti-mouse pendrin (Hafner et al, 2008; 1:1000), mouse anti-human Villin (Acris, Herford, Germany, 1:200), sheep anti-human albumin (Biotrend Chemikalien, Cologne, Germany, 1:100) mouse anti-human β-actin (Sigma–Aldrich, St. Louis, MO, USA, 1:50,000).

*Secondary antibodies for immunofluorescence*: goat anti-rabbit coupled with Alexa 555 (Invitrogen, Karlsruhe, Germany 1:2000), goat anti-chicken coupled to Alexa 488 dye (Invitrogen, Karlsruhe, Germany, 1:800), donkey anti-guinea pig Cy5 (Jackson laboratories, 1:800), DAPI (Invitrogen, Karlsruhe, Germany).

*Secondary antibodies for Western Blotting*: goat anti-rabbit (Bio-Rad Laboratories, Hercules, CA, USA, 1:10,000), goat anti-mouse (Bio-Rad Laboratories, Hercules, CA, USA, 1:10000), goat anti-guinea pig (Jackson Laboratories, West Grove, PA, USA, 1:10000).

### Statistics

Unless otherwise indicated, statistical significance was tested with Student's *t* test and the mean values are given with SEM.

## Supplementary material

Detailed facts of importance to specialist readers are published as “Supporting Information”. Such documents are peer-reviewed, but not copy-edited or typeset. They are made available as submitted by the authors.
