# Supplementary material for: A mouse model for distal renal tubular acidosis reveals a previously unrecognized role of the V-ATPase a4 subunit in the proximal tubule
Source: EMBO Mol Med. 2012 Aug 30;4(10):1057–71. doi: 10.1002/emmm.201201527 (PMC3491836; doi:10.1002/emmm.201201527)
Supplement: Supplementary file 1 [file emmm0004-1057-SD1.pdf]

EMBO
Molecular Medicine

Manuscript EMM-2012-01527

A mouse model for distal renal tubular acidosis reveals a previously unrecognized role of the V-ATPase a4 subunit in the proximal tubule

J. Christopher Hennings, Nicolas Picard, Antje K. Hübner, Tobias Stauber, Hannes Maier, Dennis Brown, Thomas Jentsch, Rosa Vargas-Poussou, Dominique Eladari and Christian A. Hübner

Corresponding author: Christian A. Hübner, Institut für Humangenetik

Review timeline:

Submission date:	29 April 2012
Editorial Decision:	31 May 2012
Revision received:	19 June 2012
Accepted:	10 July 2012

Transaction Report:

(Note: With the exception of the correction of typographical or spelling errors that could be a source of ambiguity, letters and reports are not edited. The original formatting of letters and referee reports may not be reflected in this compilation.)

1st Editorial Decision 31 May 2012

Thank you for the submission of your manuscript to EMBO Molecular Medicine. We have now heard back from two of the three referees whom we asked to evaluate your manuscript. To avoid any further delay, as both reports are consistent, we decided to let you know of our (pre)decision ahead, so you can start on the revised manuscript.

As you can see from the reports below, both referees find the study of interest, however both suggest extensive editing and revising of figures and sections of the text.

We would welcome the submission of a revised version for further consideration and depending on the nature of the revisions, this may be sent back to the referees for another round of review. Please note that this is a pre-decision only and the final outcome for the study will depend on the last review that we will forward to you as soon as it will become available.

Revised manuscripts should be submitted within three months of a request for revision; they will otherwise be treated as new submissions, except under exceptional circumstances in which a short extension is obtained from the editor. Also, the length of the revised manuscript may not exceed 60,000 characters (including spaces) and, including figures, the paper must ultimately fit onto optimally ten pages of the journal. You may consider including any peripheral data (but not methods in their entirety) in the form of Supplementary information.

I look forward to seeing a revised form of your manuscript as soon as possible.

Yours sincerely,

Editor
EMBO Molecular Medicine

Referee #1 (Comments on Novelty/Model System):

We believe that the technical quality of the work presented in this manuscript is generally high and this manuscript would improve greatly if authors describe the conditions meticulously. This manuscript describes the role of V-ATPase subunit a4 isoform using mouse KO mutant and their findings are novel. This model system gives us an opportunity to further investigate issues that are extremely relevant to a human genetic condition and potentially their cures.

Referee #1 (Other Remarks):

This manuscript describes a novel observation showing that the vacuolar-type ATPase (V-ATPase) subunit a4 isoform has a function both in distal and proximal renal tubules. The authors have generated a4 knock-out (KO) mouse that suffers from severe acidosis and proximal tubule dysfunction. Despite a small compensatory increase in renal tubular expression of a3 isoform in the a4 KO mouse, a significant reduction of B1 expression and a defective A subunit localization in the urinary tract was observed. They have also reported a4 co-localization with pendrin at the apical side of epithelial cells in the inner ear and a4 KO mouse displaying enlarged endolymphatic fluid compartments. These results indicate a vital function of V-ATPase and the a4 isoform. The a4 KO mouse may provide us with new insights into the divergent functions of the V-ATPase and pathophysiology of the a4-related symptoms. Their studies are relevant to a severe human genetic condition and, therefore, a4 KO mouse as a model system, generated in this study is of extreme importance for the future investigation of the V-ATPase. This work may have a considerable impact in the related field and also to the more general medical field. However, extensive revisions and editing are required before this manuscript could be considered for publication.

General points:
Experiments are well planned and the logical flow of the manuscript is generally good. The immunolocalization-staining results are mostly clear and it is noted that some statistical analysis was performed. However, there is no indication whether they have repeated experiments to substantiate the statistical results. Furthermore, figures are not adequately presented and elaboration is necessary. Specifically, figure legends contain sentences that should be presented in the results and did not describe vital information on experimental conditions. It is not clear in most of the photographic results which cell/tissue type is shown and where the border of the relevant cell is. It will be very helpful if a larger view of the tissue with lower magnification is shown for the figures 2A-D, and 2H-K, 3A-B, 4A-F, 4I-L, 5D-E, and 6H-I. Also, indicate the duct, endolymphatic sac, intercalated and principal cells, and the polarity of the cells in general in the photographic results throughout to help general readers understand the system. Abbreviated names should be defined in the introduction. The following specific points must be also considered.

Specific points:

Statistical analysis has asterisks in the figure indicating significance and this should be noted in the legend. An acidic urinary pH 6.66 was observed in the a4 KO mouse model system (Table I). However, the patients with defective a4 showed urinary pH of 7.47 which is apparently higher than the neutral pH. As there is no WT (normal human individual) data are shown as controls, it is hard to assess if the pH of the patient urines can be considered acidic. Please also specify the term acidosis.

pp.8 l.23~
"the expression and/or assembly of the V-ATPase complex are severely perturbed ~". Western blotting and Co-IP or RT-PCR analysis is necessary to examine the expression of other subunits of this ATPase in order to substantiate this claim.
pp.9 l.13~
The entire paragraph should be moved to "Discussion".
pp.30 Figure 2
The set of these figures mostly showed localization of V-ATPase subunits in the intercalated cells of the cortical collecting duct and the metabolic result is only 2 out of 11 panels. It would be helpful if

the title of this figure legend is given accordingly.
pp.30 l.3.
(A) is showing a section (singular) and therefore this should not be section(s).
pp.30 l.4.
(B) is showing a section (singular) from a mouse and should not be mice.
pp.30 l.10
(H-I) It is not clear where the borders of cells shown in these figures are. Nuclear staining is required in order to clarify where the cells are.
pp.30 l.11~
These sentences are result and should be included in the text. (J-K) It is also not clear what we are looking at. These electron micrographs should be presented with that of lower magnification to show what cells/tissues are shown. Qualities of the electron micrographs should be improved.
pp.31 Figure 3
It is not clear where the border of the cell is. Nuclear staining may be required. The authors are giving one example of this photograph and conclude that the numbers of intracellular vesicles and mitochondria is decreased in intercalated cells of a4 KO mice. This is not convincing at all. This claim should be substantiated by numbers of these organelles counted in the WT and KO and with statistical results.
pp.31 l.7
The vesicles and mitochondria in the ICs should be indicated.
pp.32 Figure 4
The title of this figure legend should be more specific and in which cells lysosomal material accumulates should be indicated (e.g. arrowheads).
pp.32 l.11
(I-J) is showing representative a result of a mouse. Therefore, should be singular.
pp.32 l.12
The blush border membrane (K-L) is not clear. This should be clarified by showing electron micrograph of the full view of the cell with blush order membrane.
pp.33 l.4
It is not described exactly where the "Expression of NaPi-IIa is strongly decreased".
pp.33 l.7
The sentence including, "~increased protein levels in spot urine samples from a4 KO mice." should be in the results.
pp.33 l.10
(D-E) may be showing some sort of cells but it is not clear where readers should focus. Please specify the cell/tissue type and their conditions.

Referee #2 (Other Remarks):

The manuscript "A mouse model for distal renal tubular acidosis reveals a previously unrecognized role of the V-ATPase a4 subunit in the proximal tubule" by Hennings et al. provides new and important information to understand the development of dRTA. Using KO mice as a model system the authors presented data which suggest that the severe etiopathology of the a4-related dRTA compared to the B1-related form may be depend on the absence of additional expression of a4 in the proximal tubule as compared to healthy mice. The deletion of subunit a4 not only led to a defect in acid secretion in the distal tubule but apparently also to a deficiency of the endocytotic pathway in the proximal tubule. In contrast to the deletion of the B1 subunit performed in a previous study by Karet and co-workers, the deletion of a4 in mice led to deafness which is also a symptom of a group of human patients suffering from dRTA. Finally the authors reanalyze a data set from human patients suffering from dRTA caused by mutations in a4 or B1 for differences and found that a4-related dRTA seemed to be more severely.
The performed experiments and the presented results are very convincing, however from my point of view some improvement of the manuscript especially in the Discussion is necessary. Detailed suggestions will be given in the following sections.

Abstract and introduction:
For those readers who are not familiar with V-ATPases it would be very helpful to include a more detailed description of the general structure of V-ATPases e. g. that the B1 subunit is part of the

catalytic V1 domain which is oriented to the cytosol and that the subunit a4 is an integral part of the membrane embedded VO domain. Especially the fact that subunit a4 is an integral membrane protein may be of interest for the interpretation of the presented data. A short overview on the possible V- isoforms for the B and a subunit of the V-ATPase would also be beneficial for understanding of some of the interpretations.

Results:
Page 6, last line:
"Normally the V-ATPase is translocated from the cytoplasm to the apical plasma membrane of type A-ICs upon acidosis, whereas it is translocated from the basolateral plasma membrane to the cytoplasm in type B-ICs." Please add an adequate reference.
Page 7, line 3:
Although the immune signal of subunit A in KO mice is "diffusely cytoplasmic" it seems to be in total comparably strong as in the WT, indicating that the V-ATPase or the V1 complex is not attached to the plasma membrane (Fig. 2H-I), phenomena well known regulatory mechanisms of the V-ATPase. This equal signals are in contrast to the drastically reduced signal observed for subunit B in Western blots (Fig. 2G) and for subunit A in the electron micrographs (Fig. 2J-K). The authors should comment this observation. Is the reduction of subunit B really a down regulation or is it more likely the result of a degradation process?

Discussion:
Meanwhile there are several reports indicating that the membrane specific targeting of the V-ATPase is regulated by the different isoforms of the a subunit, that subunit a is essential for assembly and function of the V-ATPase, and that the knock down or deletion of one of these isoforms could not be simply compensated by other isoforms. In addition there are also reports suggesting a second important function of the a subunit of the V-ATPase, beside the proton transport, in membrane fusion which also may provide the possibility for interpretation of the result that there is an accumulation of lysosomal compartments. The integration of these recent findings in V-ATPase research in the discussion is very important and will improve the quality and impact of the manuscript.
Legends to Fig. 1C, 2G, 5A and sup. 4C:
Please indicate the origin of the multiple lanes. Do they show the signal of "individual mice" or "independent preparations"?

1st Revision - authors' response 19 June 2012

Point-to-point response to the referees' comments

Referee #1 (Comments on Novelty/Model System):
We believe that the technical quality of the work presented in this manuscript is generally high and this manuscript would improve greatly if authors describe the conditions meticulously. This manuscript describes the role of V-ATPase subunit a4 isoform using mouse KO mutant and their findings are novel. This model system gives us an opportunity to further investigate issues that are extremely relevant to a human genetic condition and potentially their cures.

We are pleased to hear the quality and novelty of our findings. We also provide now more details about the experimental conditions as indicated below.

Referee #1 (Other Remarks):
This manuscript describes a novel observation showing that the vacuolar-type ATPase (V-ATPase) subunit a4 isoform has a function both in distal and proximal renal tubules. The authors have generated a4 knock-out (KO) mouse that suffers from severe acidosis and proximal tubule dysfunction. Despite a small compensatory increase in renal tubular expression of a3 isoform in the a4 KO mouse, a significant reduction of B1 expression and a defective A subunit localization in the urinary tract was observed. They have also reported a4 colocalization with pendrin at the apical side of epithelial cells in the inner ear and a4 KO mouse displaying enlarged endolymphatic fluid compartments. These results indicate a vital function of V-ATPase and the a4 isoform. The a4 KO

mouse may provide us with new insights into the divergent functions of the VATPase and pathophysiology of the a4-related symptoms. Their studies are relevant to a severe human genetic condition and, therefore, a4 KO mouse as a model system, generated in this study is of extreme importance for the future investigation of the V-ATPase. This work may have a considerable impact in the related field and also to the more general medical field. However, extensive revisions and editing are required before this manuscript could be considered for publication.

General points:
Experiments are well planned and the logical flow of the manuscript is generally good. The immunolocalization-staining results are mostly clear and it is noted that some statistical analysis was performed.

Thank you.

However, there is no indication whether they have repeated experiments to substantiate the statistical results.

We now provide the details how often the experiments have been repeated in the respective results section or it is stated in *Material and methods* (e.g., immunofluorescence p.16 l.37, counting of IC vesicles and mitochondria p.17, quantification of albumin in urine samples p.17) or in the respective figure legends.

Furthermore, figures are not adequately presented and elaboration is necessary. Specifically, figure legends contain sentences that should be presented in the results and did not describe vital information on experimental conditions.

Most figures (Figure 1, 2, 3, 4, 5 and 6) have now been revised and we hope that in the current version the figures are easier to read. In figure 1, e.g., we included a cartoon of the V-ATPase structure as requested by referee 2 (new Figure 1A). We included data from additional Western blots in Figure 2. The quantification of vesicles and mitochondria in ICs requested by referee 1 is now included in figure 3. We transferred details from the figure legends to the results section while including the details of the statistical analyses to the legends.

It is not clear in most of the photographic results which cell/tissue type is shown and where the border of the relevant cell is. It will be very helpful if a larger view of the tissue with lower magnification is shown for the figures 2A-D, and 2H-K, 3AB, 4A-F, 4I-L, 5D-E, and 6H-I. Also, indicate the duct, endolymphatic sac, intercalated and principal cells, and the polarity of the cells in general in the photographic results throughout to help general readers understand the system.

In the revised figures cell and tubule boundaries are symbolized by dashed lines and the lumen of tubules is indicated by an asterisk (Figure 2, 3, 4, and 5). Some requested items like overviews have now been included into the supporting information (supporting information figures 1, 4, 5, and 7). In Figure 6 we also indicate the endolymphatic sac and duct as requested.

Abbreviated names should be defined in the introduction.

This has been done as suggested.

The following specific points must be also considered.
Specific points:
Statistical analysis has asterisks in the figure indicating significance and this should be noted in the legend.

Thank you. This has been included to all figure legends.

An acidic urinary pH 6.66 was observed in the a4 KO mouse model system (Table I). However, the patients with defective a4 showed urinary pH of 7.47 which is apparently higher than the neutral pH. As there is no WT (normal human individual) data are shown as controls, it is hard to assess if the pH of the patient urines can be considered acidic.

Because the pH of the urine normally strongly depends on the systemic pH, it is difficult to compare the absolute urine pH values. Normally urine pH varies between pH 8.0 to 5.5 depending on the net acid or alkali load content of the food both in humans as in rodents. Under the stimulus of systemic acidosis, like observed here in our mouse model or in the patients analyzed in this study, urine pH is expected to be maximally decreased beyond pH 5.5. The inability to lower the urine pH below 5.5 despite of systemic acidosis thus suggests a severe distal acidification defect. This is further supported by the observation that WT and KO mice exhibit comparable ammonium excretion levels despite of strong acidosis. This important background information is now also given in the results section (p. 6, last paragraph and p. 8, second paragraph).

Please also specify the term acidosis.

As requested we now specify the term acidosis as a decrease in blood pH below 7.38 in the results section (page 6, last paragraph).

pp.8 l.23~
"the expression and/or assembly of the V-ATPase complex are severely perturbed ~". Western blotting and Co-IP or RT-PCR analysis is necessary to examine the expression of other subunits of this ATPase in order to substantiate this claim.

As suggested, we performed additional Western blot analyses of kidney lysates for the E1 and the A subunit of the V1 complex. Supporting a down-regulation of the V-ATPase complex, both of these subunits were reduced in a4 KO mice. This is now shown in Figure 2G. The wording has also been specified (p.7, second paragraph).

pp.9 l.13~
The entire paragraph should be moved to "Discussion".

Has been done as suggested (now on page 12, second paragraph).

pp.30 Figure 2
The set of these figures mostly showed localization of V-ATPase subunits in the intercalated cells of the cortical collecting duct and the metabolic result is only 2 out of 11 panels. It would be helpful if the title of this figure legend is given accordingly.

We changed the title accordingly: “Localization of the a4 subunit in the distal tubule and metabolic acidosis in a4 KO mice”.

pp.30 l.3.
(A) is showing a section (singular) and therefore this should not be section(s).

Thank you. This has been corrected accordingly.

pp.30 l.4.
(B) is showing a section (singular) from a mouse and should not be mice.

Thank you. This has been corrected accordingly as well.

pp.30 l.10
(H-I) It is not clear where the borders of cells shown in these figures are. Nuclear staining is required in order to clarify where the cells are.

We performed additional stainings (Supporting information figure 4A-D), however, the old staining still had the best “signal-to-noise” ratio. We increased the contrast and brightness, so that the nucleus, which is not stained, can be more easily distinguished from the surrounding cytoplasm. For clarity the basolateral and apical borders of the tubular epithelium are indicated by dotted lines and the lumina are marked with an asterisk.

pp.30 l.11~
These sentences are result and should be included in the text.

© European Molecular Biology Organization

Thank you. This has been corrected as well.

(J-K) It is also not clear what we are looking at. These electron micrographs should be presented with that of lower magnification to show what cells/tissues are shown. Qualities of the electron micrographs should be improved.

The cryo EM images show the apical cell pole of ICs. For orientation we now provide also lower magnification images showing complete collecting duct cross sections (Supporting information figure 5).
We agree that the tissue preservation is better in plastic embedded tissue samples. However, the immunogold labeling only worked on cryosections at the expense of some subcellular details.

pp.31 Figure 3
It is not clear where the border of the cell is. Nuclear staining may be required. The authors are giving one example of this photograph and conclude that the numbers of intracellular vesicles and mitochondria is decreased in intercalated cells of a4 KO mice. This is not convincing at all. This claim should be substantiated by numbers of these organelles counted in the WT and KO and with statistical results.

The basolateral borders of collecting ducts are now indicated by a dashed line. In the electron microscopy images the lateral cell borders of ICs are now indicated by a dashed line. As suggested by the reviewer we counted the vesicles and the mitochondria in cross-sections of several ICs. While vesicles were significantly reduced in KO ICs, the number of mitochondria was unchanged (Figure 3F and G).

pp.31 l.7
The vesicles and mitochondria in the ICs should be indicated.

Thank you. This has now been done.

pp.32 Figure 4
The title of this figure legend should be more specific and in which cells lysosomal material accumulates should be indicated (e.g. arrowheads).

We changed the title to "Accumulation of lysosomal material in proximal tubules of a4 KO mice."

pp.32 l.11
(I-J) is showing representative a result of a mouse. Therefore, should be singular.

Thank you. This has been corrected.

pp.32 l.12
The brush border membrane (K-L) is not clear. This should be clarified by showing electron micrograph of the full view of the cell with brush order membrane.

The overviews are presented as supporting information figure 7.

pp.33 l.4
It is not described exactly where the "Expression of NaPi-IIa is strongly decreased".

NaPi-IIa is strongly decreased in the brush border of proximal tubule cells, since it is almost exclusively expressed in the brush border of the proximal tubule. This has now also been specified in the results section and the figure legend (page 9 and Figure 5).

pp.33 l.7
The sentence including, "~increased protein levels in spot urine samples from a4 KO mice." should be in the results.

This has been changed as suggested.

pp.33 l.10
(D-E) may be showing some sort of cells but it is not clear where readers should focus. Please specify the cell/tissue type and their conditions.

We have specified the tissue and cell type in the figure legend and think that the difference for albumin deposits (stained in green and absent in WT) in proximal tubule cells is convincing.

Referee #2 (Other Remarks):
The manuscript "A mouse model for distal renal tubular acidosis reveals a previously unrecognized role of the V-ATPase a4 subunit in the proximal tubule" by Hennings et al. provides new and important information to understand the development of dRTA. Using KO mice as a model system the authors presented data which suggest that the severe etiopathology of the a4-related dRTA compared to the B1-related form may be depend on the absence of additional expression of a4 in the proximal tubule as compared to healthy mice. The deletion of subunit a4 not only led to a defect in acid secretion in the distal tubule but apparently also to a deficiency of the endocytotic pathway in the proximal tubule. In contrast to the deletion of the B1 subunit performed in a previous study by Karet and co-workers, the deletion of a4 in mice led to deafness which is also a symptom of a group of human patients suffering from dRTA. Finally the authors reanalyze a data set from human patients suffering from dRTA caused by mutations in a4 or B1 for differences and found that a4-related dRTA seemed to be more severely. The performed experiments and the presented results are very convincing, however from my point of view some improvement of the manuscript especially in the Discussion is necessary. Detailed suggestions will be given in the following sections.

Abstract and introduction:
For those readers who are not familiar with V-ATPases it would be very helpful to include a more detailed description of the general structure of V-ATPases e. g. that the B1 subunit is part of the catalytic V1 domain which is oriented to the cytosol and that the subunit a4 is an integral part of the membrane embedded VO domain.

We agree and extended the description of the basic structure of the V-ATPase (Introduction, page 4, first and third paragraph) and now also include a cartoon of the structure as new Figure 1A.

Especially the fact that subunit a4 is an integral membrane protein may be of interest for the interpretation of the presented data. A short overview on the possible V- isoforms for the B and a subunit of the V-ATPase would also be beneficial for understanding of some of the interpretations.

This information is now given in the introduction.

Results:
Page 6, last line:
"Normally the V-ATPase is translocated from the cytoplasm to the apical plasma membrane of type A-ICs upon acidosis, whereas it is translocated from the basolateral plasma membrane to the cytoplasm in type B-ICs."

A reference is now given (page 7, Schwartz et al., 1985).

Page 7, line 3:
Although the immune signal of subunit A in KO mice is "diffusely cytoplasmic" it seems to be in total comparably strong as in the WT, indicating that the V-ATPase or the V1 complex is not attached to the plasma membrane (Fig. 2H-I), phenomena well known regulatory mechanisms of the V-ATPase. The equal signals are in contrast to the drastically reduced signal observed for subunit B in Western blots (Fig. 2G) and for subunit A in the electron micrographs (Fig. 2J-K). The authors should comment this observation. Is the reduction of subunit B really a down regulation or is it more likely the result of a degradation process?

It is difficult to compare staining intensities when the location of the antigen is changed: Subunit A is concentrated apically in a narrow band in WT (H), and diffuse in KO (I). Although acquired with the same microscope settings, all we can say with certainty is that the location is altered. For

quantification we have now performed additional Western blots as requested including the A subunit and found significantly decreased expression levels for the A subunit in the a4 KO tissue (Figure 2G).

Discussion:
Meanwhile there are several reports indicating that the membrane specific targeting of the V-ATPase is regulated by the different isoforms of the a subunit, that subunit a is essential for assembly and function of the V-ATPase, and that the knock down or deletion of one of these isoforms could not be simply compensated by other isoforms. In addition there are also reports suggesting a second important function of the a subunit of the V-ATPase, beside the proton transport, in membrane fusion which also may provide the possibility for interpretation of the result that there is an accumulation of lysosomal compartments. The integration of these recent findings in V-ATPase research in the discussion is very important and will improve the quality and impact of the manuscript.

We are grateful for this important suggestion. We have extended the discussion accordingly (pages 11, 12 and 13) and included the appropriate references (Peters et al., 2001; Bayer et al., 2003; Hiesinger et al., 2005).

Legends to Fig. 1C, 2G, 5A and sup. 4C: Please indicate the origin of the multiple lanes. Do they show the signal of "individual mice" or "independent preparations"?

In general, each line is from an individual mouse. This information has now been added to the respective figure legends (Figure 1D, 2G and 5A; supporting information figure 8C).
