# Supplementary material for: A mouse model for distal renal tubular acidosis reveals a previously unrecognized role of the V-ATPase a4 subunit in the proximal tubule
Source: EMBO Mol Med. 2012 Aug 30;4(10):1057–71. doi: 10.1002/emmm.201201527 (PMC3491836; doi:10.1002/emmm.201201527)
Supplement: Supplementary file 2 [file emmm0004-1057-SD2.pdf]

Supporting information

Table of contents

Supporting information figure 1 .... **page 2**
Supporting information figure 2 .... **page 3**
Supporting information figure 3 .... **page 3**
Supporting information figure 4 .... **page 4-5**
Supporting information figure 5 .... **page 5**
Supporting information figure 6 .... **page 5**
Supporting information figure 7 .... **page 6**
Supporting information figure 8 .... **page 7**

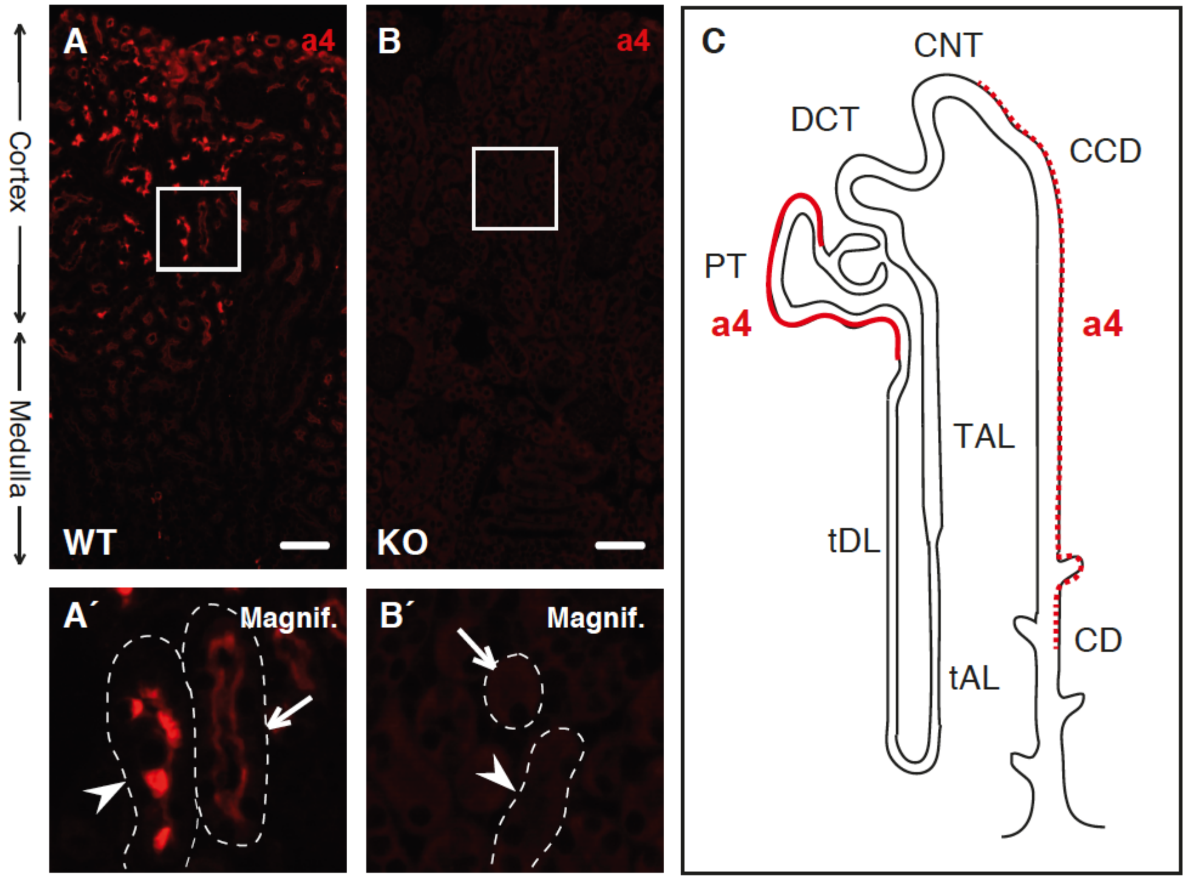

Supporting information figure 1: The basolateral membrane of the epithelium of some randomly selected tubules are indicated by dashed lines. **(A)** Low and high magnifications of a WT kidney section stained for the a4 subunit reveals moderate apical expression of the a4 subunit in the proximal tubule (asterisk) compared to the strong apical expression in intercalated cells (arrowheads). **(B)** In the a4 KO specific signals for the a4 subunit were absent. Scale bars: 60 µm. **(C)** Schematic cartoon showing the expression of the a4 subunit along the nephron. The a4 subunit is expressed in the proximal tubule (PT, red line) and a subset of cells of the cortical collecting duct (CCD, dashed line) and medullary collecting duct epithelium (CD, dashed line). Abbreviations: thin descending limb (tDL), thin ascending limb (tAL), thick ascending (TAL), distal convoluted tubule (DCT), connecting tubule (CNT).

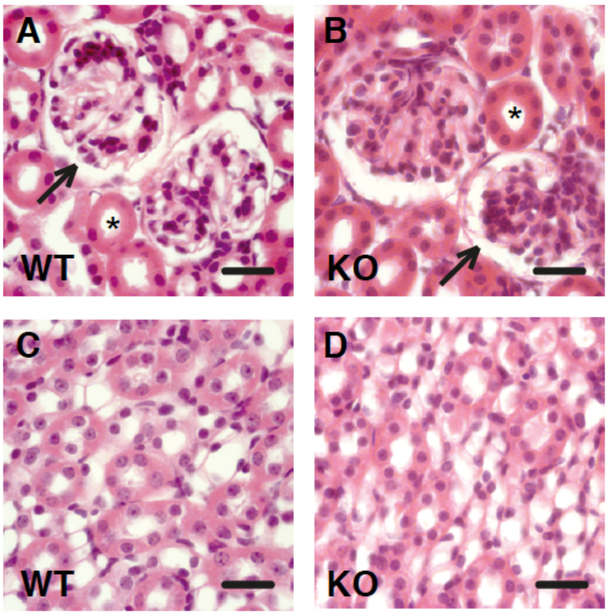

Supporting information figure 2: (A-B) HE histology reveals no obvious morphological changes of glomeruli and cortical tubules (asterisk) in an a4 KO compared to a WT mouse. **(C-D)** HE histology of the medulla also shows no gross differences between genotypes. Scale bars: 20 µm.

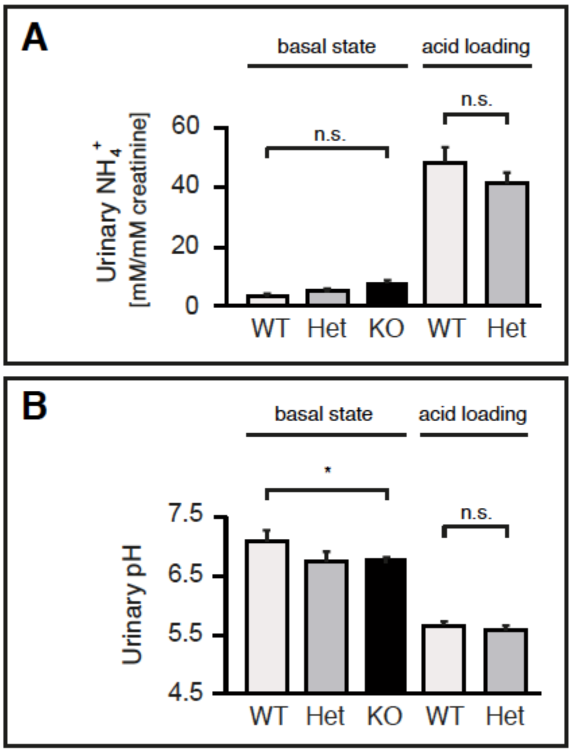

Supporting information figure 3: (A) Urinary ammonium during basal state and after acid loading with NH_4Cl. As expected, ammonium excretion is strongly increased following acid loading, but no significant differences between WT and *Atp6v0a4*$^{+/-}$ mice were observed (Basal state values listed in Table I, WT: 48.9 ± 4.9 mM/mM creatinine (n = 9), Het: 42.4 ± 2.4 mM/mM creatinine; n = 7). **(B)** Urinary pH during basal state and after acid loading with NH_4Cl. Following acid loading urinary pH decreased, but no significant differences between WT and *Atp6v0a4*$^{+/-}$ mice were observed (Basal state values listed in Table I, WT: 5.60 ± 0.04 (n = 8), Het: 5.51 ± 0.08; n = 4).

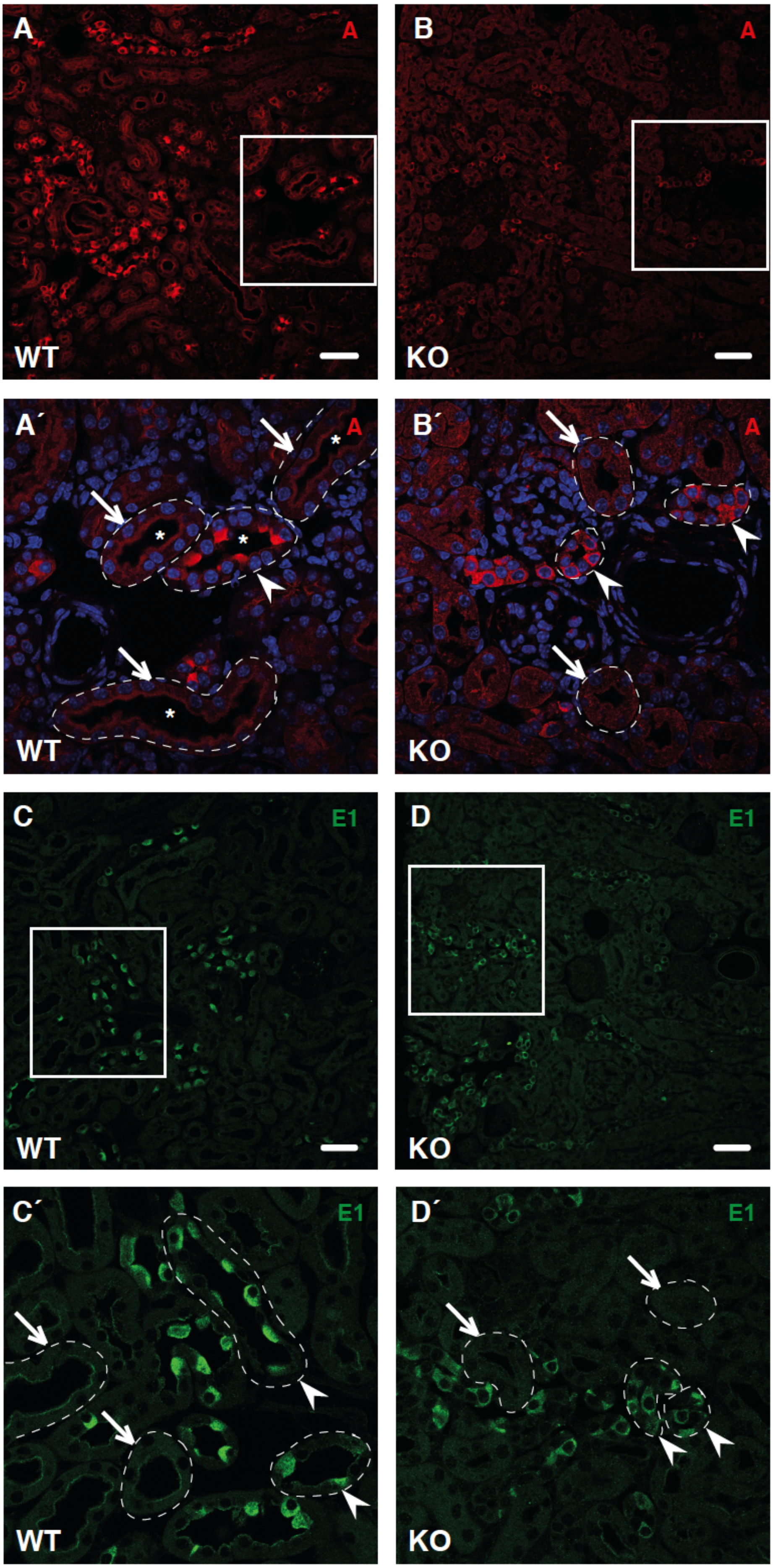
A
A
WT
B
A
KO
A´
A
WT
B´
A
KO
C
E1
WT
D
E1
KO
C´
E1
WT
D´
E1
KO

Supporting information figure 4: The basolateral border of the epithelium of some randomly selected tubules is indicated by dotted lines and the lumen with an asterisk. Arrows indicate the proximal tubules, arrowheads collecting ducts and asterisks the tubular lumen. **(A-B)** Immunofluorescence for the A subunit (red) at low (A) and high (A') magnification of a WT and a4 KO kidney section. **(C-D)** Immunofluorescence for the E1 subunit (green) at low (C) and high (C') magnification of a WT and a4 KO kidney section. Scale bars: 50 µm.

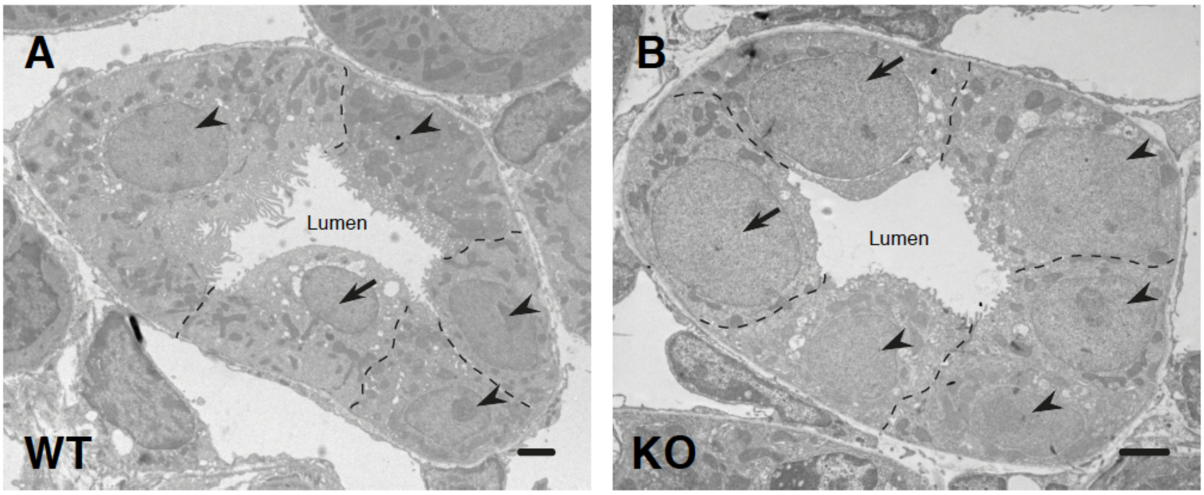

Supporting information figure 5: (A-B) Electron microscopy overview of a cross-sectioned collecting duct from an *Atp6v0a4*$^{+/+}$ and a *Atp6v0a4*$^{-/-}$ mouse. Cell borders are indicated by a dashed line. Highlighted are principal cells (arrows) and intercalated cells (arrowheads). Scale bars: 4 µm.

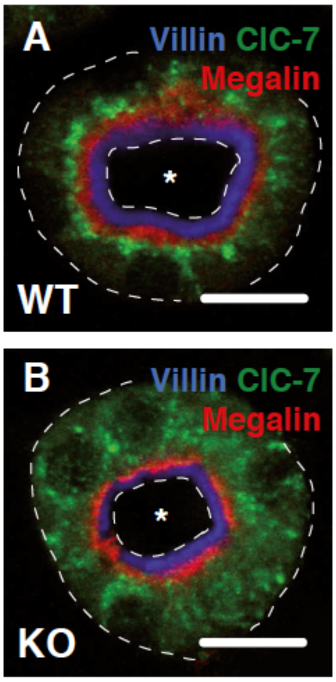

Supporting information figure 6: (A-B) a4 KO mice show more, and more broadly distributed lysosomal vesicles, as demonstrated by ClC-7-positive (green) intracellular vesicles. Villin (blue), megalin (red). Scale bars: 10 µm.

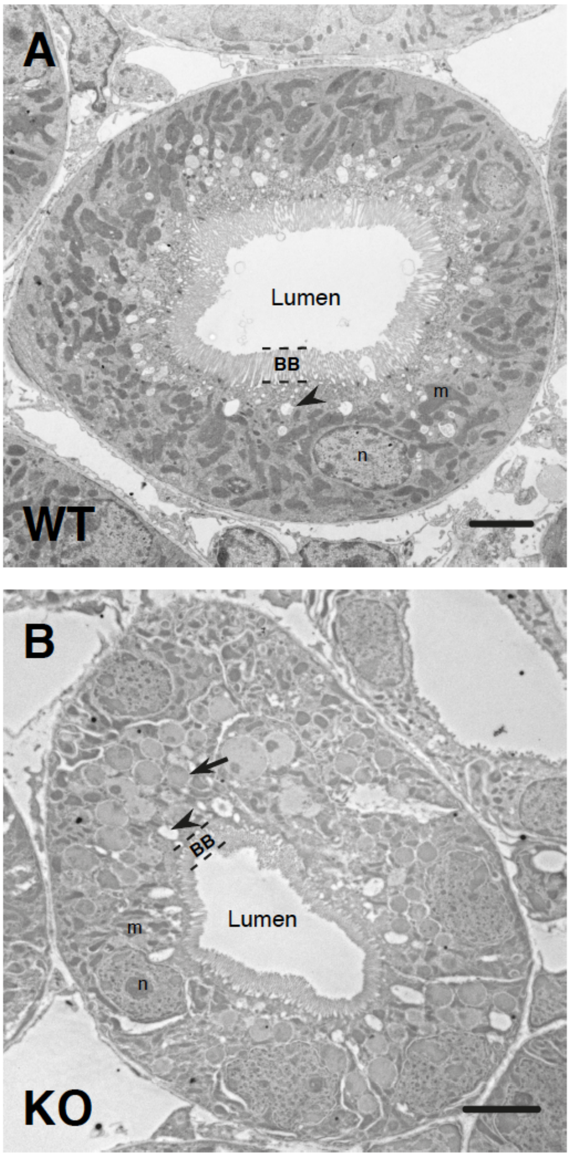

Supporting information figure 7: (A-B) Electronmicoscopy of a proximal tubule cross section from an *Atp6v0a4*$^{+/+}$ and *Atp6v0a4*$^{-/-}$ mouse. The borders of the brush border (BB) is indicated by dashed lines, intracellular vesicles by arrowheads. In *Atp6v0a4*$^{-/-}$ mice lysosome-like structures are indicated by arrows. Other subcellular structures highlighted: nucleus (n) and mitochondria (m). Scale bars: 4 µm.

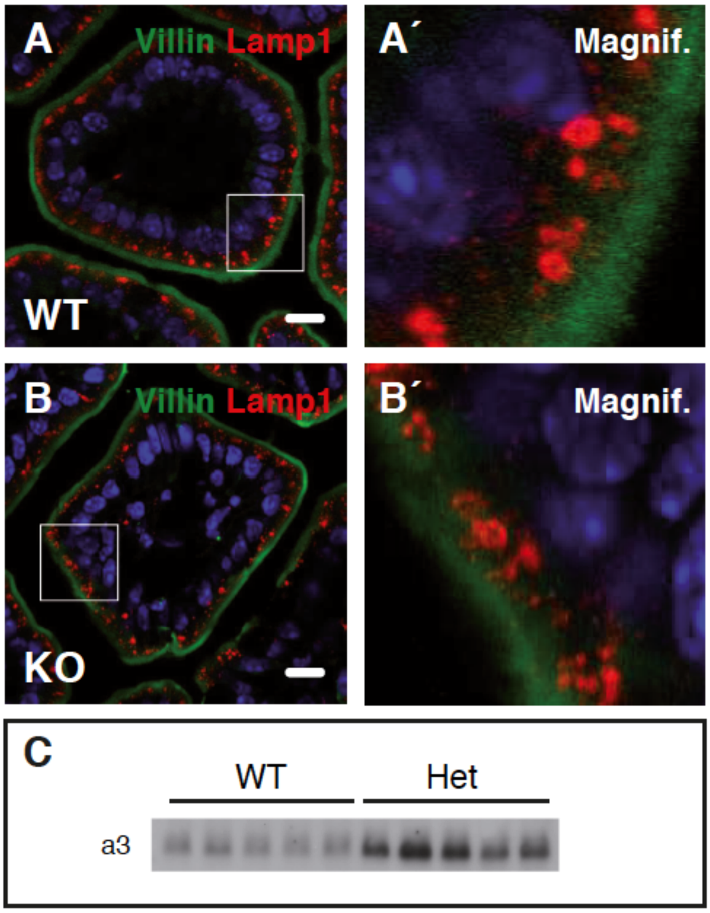

Supporting information figure 8: (A-B) Analysis of the lysosomal compartment of enterocytes of the small intestine of an *Atp6v0a4*$^{-/-}$ and WT mouse. No obvious differences in the lysosomal compartment are noted between WT and *Atp6v0a4*$^{-/-}$ mice despite pronounced dRTA with systemic acidosis in *Atp6v0a4*$^{-/-}$ mice. Scale bars: 10 µm. **(C)** Western Blot analysis of a3 expression in *Atp6v0a4*$^{+/-}$ and WT mice shows an upregulation of the a3 subunit in heterozygous mice (WT: 1.0 ± 0.05, KO: 2.78 ± 0.34, Student´s t test: $p < 0.001$).
